# *F13B* regulates angiogenesis and tumor progression in hepatocellular carcinoma via the HIF-1α/VEGF pathway

**DOI:** 10.17305/bb.2024.10794

**Published:** 2024-09-10

**Authors:** Dong Jiang, Zhi Qi, Zhi-ying Xu, Yi-ran Li

**Affiliations:** 1 Department of Ultrasound, Eastern Hepatobiliary Surgery Hospital, The Third Affiliated Hospital of Naval Medical University, Shanghai, China; 2 Department of Neurology, Eastern Hepatobiliary Surgery Hospital, The Third Affiliated Hospital of Naval Medical University, Shanghai, China; 3 Department of Hepatic Surgery IV, Shanghai Eastern Hepatobiliary Surgery Hospital, Third Affiliated Hospital of Naval Medical University, Shanghai, China

**Keywords:** *F13B*, HIF-1α/VEGF pathway, hepatocellular carcinoma, angiogenesis, tumor progression

## Abstract

Hepatocellular carcinoma (HCC) is a highly aggressive malignant tumor with a poor prognosis. This research aimed to investigate the role of *F13B* in HCC and its underlying mechanisms. Through comprehensive bioinformatics analysis of the GSE120123 and The Cancer Genome Atlas (TCGA)-Liver hepatocellular carcinoma (LIHC) datasets, we identified 220 overlapping prognosis-related genes. Eight key genes, including the previously unreported *CCDC170* and *F13B* in HCC, were identified through Least Absolute Shrinkage and Selection Operator (LASSO)-Cox regression analysis. *F13B* emerged as a significant prognostic factor in HCC, warranting further investigation in subsequent analyses. *In vitro* experiments showed that *F13B* expression was notably reduced in HCC cell lines and tissues, particularly in Huh-7 and SMMC-7721 cells. Overexpression of *F13B* inhibited cell invasion, migration, and proliferation, while its knockdown produced the opposite effect. A lactate dehydrogenase (LDH) activity assay in human umbilical vein endothelial cells (HUVECs) demonstrated that *F13B* overexpression reduced vascular endothelial growth factor (VEGF)-induced cytotoxicity, whereas knockdown increased it. Further analysis revealed that *F13B* negatively regulates *VEGFA* expression, affecting HUVEC proliferation. In HUVECs, *F13B* overexpression reversed VEGF-induced upregulation of key angiogenesis markers, including phospho-VEGF receptor 2 (p-VEGFR2), matrix metalloproteinase-2 (MMP-2), matrix metalloproteinase-9 (MMP-9), as well as AKT/mTOR signaling proteins, phospho-Akt (p-AKT), and phospho-mTOR (p-mTOR). Additionally, *F13B* negatively regulated *VEGFA* and hypoxia-inducible factor 1 A (*HIF1A*) under hypoxic conditions, counteracting the hypoxia-induced increase in cell viability. These findings suggest that *F13B* regulates angiogenesis through the HIF-1α/VEGF pathway and plays a crucial role in HCC progression. Our results highlight the potential of *F13B* as a therapeutic target in HCC, providing novel insights into the molecular mechanisms of HCC and its prognostic significance.

## Introduction

Hepatocellular carcinoma (HCC) is an extremely aggressive cancer characterized by irreversible changes in the liver, often triggered by factors, such as drug-induced hepatitis, viral hepatitis B, idiopathic hepatitis, and local tumors [1, 2]. The progression of HCC to advanced stages is frequently accompanied by severe complications, including hepatic encephalopathy, upper gastrointestinal bleeding, massive ascites, rupture and bleeding of the tumor, metastatic pleural effusion, infections, fever, liver and kidney failure, and respiratory failure [3, 4]. These complications contribute significantly to the morbidity and mortality associated with HCC, posing a substantial threat to public health and placing a heavy burden on affected families [5]. Moreover, the treatment of HCC faces many challenges, including resistance to chemotherapy and radiotherapy, as well as a lack of effective early diagnostic and prognostic markers. In recent years, with the development of omics technologies, researchers have begun to identify molecular pathways associated with the development of HCC and potential therapeutic targets. However, there are still many unresolved issues in the field of HCC research, including the molecular mechanisms of disease progression, the role of the tumor microenvironment, and how to more effectively translate basic research findings into clinical applications. Therefore, there is an urgent need for further research to elucidate the latent molecular pathways involved in the development and progression of HCC, in order to develop more effective prevention, diagnosis, and treatment strategies.

Vascular endothelial growth factor A (*VEGFA*) is a protein crucial for angiogenesis, promoting blood vessel formation and endothelial cell proliferation [6]. In HCC, *VEGFA* is essential to the development and spread of tumors by promoting angiogenesis, facilitating tumor vascularization, and enhancing tumor cell survival [7]. Zucman-Rossi et al. [8] demonstrated that amplification of *VEGFA* on chromosome 6p21 is associated with HCC progression, and they also identified other genetic alterations such as FGF19/CNND amplification. These findings suggest potential therapeutic targets for advanced HCC and advocate for precision medicine approaches. Furthermore, Zhao et al. [9] demonstrated that downregulation of microRNA-205 in HCC inhibits cell growth and metastasis by directly targeting *VEGFA*, offering promising therapeutic prospects. Additionally, Zhang et al. [10] found that *VEGFA* shows increased expression in HCC tissues and serum, correlating with α-fetoprotein levels, thereby enhancing its diagnostic potential alongside KAI1. The combined detection of *VEGFA* and KAI1 proposes a robust diagnostic marker panel, improving the efficacy of HCC diagnosis. It is critical to have a thorough grasp of the function of *VEGFA* in HCC. By elucidating the role of *VEGFA* and related pathways in HCC, new therapeutic approaches may be developed to inhibit tumor growth and metastasis, which may ultimately improve patient prognosis.

The *F13B* gene encodes the B subunit of coagulation factor XIII, which is essential for blood clot stabilization [11]. Factor XIII is a transglutaminase that cross-links fibrin, thereby strengthening and stabilizing the blood clot [12]. The B subunit acts as a carrier for the active A subunit, ensuring its stability and proper function. Mutations in the *F13B* gene can lead to Factor XIII deficiency, an uncommon bleeding disorder marked by impaired clot formation, delayed wound healing, and spontaneous bleeding [13]. Recent research has highlighted the potential involvement of *F13B* in various cancers. Yang et al. [14] identified *F13B* as one of the prominently dysregulated proteins in the urine of clear cell renal cell carcinoma (ccRCC) patients, suggesting its potential significance in ccRCC diagnosis and prognosis, although it lacked statistical significance in the final analysis. Similarly, Pietsch et al. [15] suggested a potential function for *F13B* acting as an anti-cancer gene in medulloblastoma pathogenesis, noting that it is located on chromosome 1q31-q32.1, a region exhibiting allelic loss in medulloblastomas. Additionally, Iino et al. [16] indicated a plausible association between *F13B* and colorectal cancer, particularly in the context of DNA microsatellite instability (MSI) in colorectal polyps. These studies collectively underscore the potential involvement of *F13B* in the pathogenesis of various cancers, urging further investigation into its precise mechanisms and clinical implications. Given these findings, it is crucial to explore the function of *F13B* in HCC to comprehend its possible impact on tumor behavior and patient outcomes. Through the integration of experiments with HCC cell lines and HUVECs, we aimed to explore the gene’s intrinsic effects on tumor cells and its impact on angiogenesis, a critical component of tumor progression. This dual approach is designed to gain a deeper understanding of the complex interplay between cancer cells and endothelial cells, which is essential for comprehending metastasis and developing potential therapeutic strategies.

Despite advances in understanding the pathogenesis of HCC, the precise molecular mechanisms remain elusive, highlighting the need for further research. *VEGFA* is essential for angiogenesis and has a substantial effect on the growth of HCC. Prior research has demonstrated the presence of multiple gene alterations and regulatory pathways in HCC, highlighting the potential for therapeutic interventions targeting these mechanisms [17]. The *F13B* gene has shown potential in different cancers, but its role in HCC has not been fully elucidated. In this investigation, *F13B* was recognized as a prognostic gene for HCC through integration and bioinformatics analysis. Cell experiments were employed to explore the function of *F13B* in HCC and its mechanism, focusing on its regulation of *VEGFA* and HIF-1α/VEGF pathways. We examined the expression of *F13B* in various HCC cell lines, including Huh-7, SMMC-7721, Hep3B, and MHCC97, each selected for distinct reasons that enhance our research. Our choice of Huh-7 was driven by its high transfection efficiency and stable tumor-forming capabilities, making it ideal for molecular mechanism studies [18]. The SMMC-7721 cell line, known for its high metastatic potential, represents a valuable model for understanding HCC’s aggressive nature [19]. Hep3B was included due to its widespread use in HCC research and unique genetic attributes [20]. Lastly, the MHCC97 cell line was chosen for its ability to mimic the invasive and metastatic behavior of HCC [21]. By studying the expression and functional effects of *F13B* in HCC and endothelial cells, we seek to gain insight into its potential as a therapeutic target and prognostic marker, ultimately helping to improve patient outcomes and deepen our understanding of HCC pathogenesis.

## Materials and methods

### Dataset acquisition

A comprehensive search was performed in the Gene Expression Omnibus (GEO) database using the keyword “hepatocellular carcinoma.” From this search, we selected the GSE120123 dataset, which included profiles of gene expression of six HCC samples and ten normal control samples. To further validate and comprehensively analyze our findings, we also accessed The Cancer Genome Atlas (TCGA)-Liver HCC (LIHC) database (https://cancergenome.nih.gov/). From this database, 371 primary tumor tissues and 50 normal liver tissues were downloaded. These samples served as an independent validation cohort for subsequent gene identification and analysis. The data we utilized have been processed through the HTSeq protocol, ensuring standardized gene expression measurements, which guarantee that our analysis is based on a consistent and reliable dataset.

### Differential expression and enrichment analysis

To select differentially expressed genes (DEGs) from the GSE120123 and TCGA-LIHC samples, we utilized the “limma” package (version 3.42.2). The DEG selection criteria were established as |log2 (fold change) FC| < 1 for downregulated DEGs and |log2 (fold change) FC| > 1 for upregulated DEGs, with a significance threshold of *P* < 0.01. Kyoto Encyclopedia of Genes and Genomes (KEGG) and Gene Ontology (GO) analyses were performed on the GSE120123-DEGs for enrichment using FunRich (http://www.funrich.org/). Three categories were considered in the GO analysis: Cellular Component (CC), Biological Process (BP), and Molecular Function (MF), with findings deemed noteworthy at *P* < 0.05.

### Identification of key overlapping genes among GSE120123-DEGs, TCGA-LIHC-DEGs, and LIHC prognosis-related genes

Subsequently, we utilized the Assistant for Clinical Bioinformation (https://www.aclbi.com/static/index.html#/) database to perform a comprehensive survival analysis on all genes in the TCGA-LIHC dataset, including univariate Cox regression analysis to pinpoint genes significantly linked to overall survival. This is because overall survival is an established and definitive measure in oncology research, providing a clear and direct assessment of the impact of gene expression on patient outcomes. We utilized the “Calculate and draw custom Venn diagrams” tool (https://bioinformatics.psb.ugent.be/webtools/Venn/) for the intersection analysis of prognostic genes and DEGs. This analysis included GSE120123-DEGs, TCGA-DEGs, and prognostic genes identified in the LIHC dataset, allowing us to pinpoint key overlapping genes for further investigation.

### Overall survival (OS) analysis and construction of prognostic risk model

Using the Kaplan–Meier (KM) plotter website (https://kmplot.com/), we performed OS analysis of overlapping genes and identified genes with significant results (*P* < 0.05). Next, prognostic genes were subjected to the Least Absolute Shrinkage and Selection Operator (LASSO) Cox regression analysis using the “glmnet” R package (version 4.1.3). Ten-fold cross-validation analysis was utilized to choose the characteristic gene coefficients based on the minimum value of lambda. Patients were categorized into low-risk and high-risk groups based on the expression of distinctive genes in TCGA-LIHC data sets. The survival status of patients in different risk groups and the clustering of genes were analyzed. To assess survival differences between the two groups, a log-rank test was employed for KM survival analysis. In addition, time-dependent receiver operating characteristic (ROC) analysis was used to evaluate the predictive ability of the risk model, and the area under the curve (AUC) value indicated the discriminative ability of the risk model.

### Expression and survival analysis of CCDC170 and *F13B*

In this study, SangerBox (http://sangerbox.com/) was utilized to examine the levels of two genes (CCDC170 and *F13B*) between GSE120123 tumors and normal tissues. The impact of differential expression of CCDC170 and *F13B* on progression-free survival (PFS), disease-specific survival (DSS), and disease-free interval (DFI) of LIHC patients was then analyzed through the Gene Set Cancer Analysis (GSCA; https://guolab.wchscu.cn/GSCA//#/) database. Results with *P* < 0.05 were considered statistically significant.

### Construction and validation of predictive nomograms

Uni/multivariate Cox regression analyses between *F13B* and clinicopathological parameters (age, gender, pT-stage, pTNM-stage) were constructed using the “forestplot” package (version 2.0.1). Following that, nomograms were built using the “rms” package (version 3.6.1). The nomogram provides graphical results that enable the calculation of a patient’s prognosis risk. The clinical parameters included in our nomogram were chosen based on their established prognostic relevance and availability across the patient cohort, ensuring broad applicability and clinical utility. The predictive accuracy of the nomograms was examined using calibration curves, which compare the predicted survival probabilities with the observed outcomes. A closer alignment of the predictive results with the calibration curve indicates better forecasted outcomes of the model.

### Gene set enrichment analysis (GSEA) and clinical characteristics analysis

To identify enriched pathways associated with *F13B*, we performed GSEA to identify biological pathways associated with *F13B* expression in HCC. UALCAN (http://ualcan.path.uab.edu/analysis.html), an extensive online resource for quick and easy access to TCGA cancer data, was utilized for gene expression analysis and validation. Using the UALCAN dataset, we confirmed the relative expression levels of *F13B* across different clinical characteristics, including patient age, nodal metastasis status, tumor grade, histological subtypes, and TP53 mutation status. By detecting statistically significant differences in *F13B* expression across different clinical categories, its potential relevance in various pathological conditions was analyzed.

### Cell lines and culture conditions

The Chinese Academy of Sciences Cell Bank (Shanghai, China) provided the HCC cell lines Hep3B, MHCC97, and Huh-7. The SMMC-7721 cell line was obtained from Cobioer Biosciences (Nanjing, China). These cells were cultured in RPMI 1640 medium supplemented with 1% penicillin–streptomycin and 10% fetal bovine serum (FBS) in a humidified atmosphere containing 5% CO_2_ at 37 ^∘^C. For hypoxic conditions, HCC cells were transferred to a hypoxia incubator (YCP-80/S, Superhorizon, China) set at 37 ^∘^C with 1% O_2_ and 5% CO_2_ once they reached 70%–80% confluence. Human umbilical vein endothelial cells (HUVECs) were bought from Cobioer Biosciences (Nanjing, China) and grown in endothelial cell medium enhanced with 1% endothelial cell growth supplement, 1% penicillin–streptomycin, and 5% FBS. To study the function of vascular endothelial growth factor (VEGF) in tumor angiogenesis, VEGF was applied to HUVECs in different concentrations (0, 5, 10, 15, 20 ng/mL) for 24 h.

Normal human liver cells (THLE-2) were acquired from Procell Biotech (Wuhan, China). These cells were cultured in BEGM (Bronchial Epithelial Cell Growth Medium BulletKit™; Lonza, Basel, Switzerland), supplemented with 5 ng/mL epidermal growth factor (EGF; Thermo Fisher Scientific, Waltham, MA, USA), 70 ng/mL phosphoethanolamine (Sigma-Aldrich, Saint Louis, MO, USA), 10% FBS, and 1% penicillin–streptomycin [22–24]. The culture was maintained in a 5% humidified CO_2_ incubator at 37 ^∘^C.

### Cell transfection

After being seeded in 6-well plates at a density of 2×10^5^ cells per well, the cells were cultivated for 24 h. Then, cells were transfected with 100-nM siRNA targeting *F13B*/*VEGFA* (si-*F13B* #1 sense: CCTGTAGGAAAGAACATGAAA, si-*F13B* #2 sense: CCAAGATGTATTCCAAGACAA, si-*VEGFA* sense: AGGGCAGAATCATCACGAAGT), overexpression *F13B*/*VEGFA* (over-*F13B*, over-*VEGFA*) plasmids, control siRNA (si-NC or over-NC) using Lipofectamine 3000, respectively. After 48 h, cells were harvested for RNA extraction.

### Quantitative real-time PCR (qRT-PCR)

TRIzol reagent (Invitrogen) was used to extract total RNA from the cells, following the manufacturer’s instructions. A PrimeScript RT kit (TaKaRa) was employed to create complementary DNA (cDNA) from the extracted RNA. SYBR Green PCR Master Mix (TaKaRa) was utilized to conduct quantitative real-time PCR (qRT-PCR) using an ABI 7500 Real-Time PCR System. As an internal control, gene expression levels were normalized to GAPDH. Target gene relative expression was assessed by the 2^-ΔΔCT^ method. The primer sequences used for qRT-PCR are listed in Table 1.

**Table 1:** Primer sequences for qRT-PCR

**Target**	**Direction**	**Sequence (5′-3′)**	**Length (bp)**	**Annealing temperatures (Tm)**
*F13B*	Forward	GACCACACATT TTGCATGGTGA	154	59.96
*F13B*	Reverse	GACAGAGTGCT TTGTCTTGGA		58.16
*VEGFA*	Forward	TCCTGGAGCGT GTACGTTG	177	59.71
*VEGFA*	Reverse	TAACTCAAGCT GCCTCGCCT		61.54
*VEGF*	Forward	CTGTCTAATGC CCTGGAGCC	124	60.18
*VEGF*	Reverse	ACGCGAGTCTG TGTTTTTGC		59.97
*HIF1A*	Forward	TTGATGGGATA TGAGCCAGA	128	55.67
*HIF1A*	Reverse	TGTCCTGTGGT GACTTGTCC		59.53
*Cyclin B1*	Forward	GCCAGTGCCAGAGCCAG AAC	103	63.70
*Cyclin B1*	Reverse	CATTGGGCTTGGAGAGG CAGTATC		63.32
*Cyclin D1*	Forward	GCCCTCGGT GTCCTACT TCAAATG	111	63.47
*Cyclin D1*	Reverse	TCCTCCTCG CACTTCTG TTCCTC		63.80
*CDC2*	Forward	ACAGGTCAAGTGGTAGC CATGA	139	61.36
*CDC2*	Reverse	GCATAAGCA CATCCTGA AGACTGAC		61.93
*CDC25*	Forward	GAAAGAGATAG CAGTGAACCAG GG	98	60.92
*CDC25*	Reverse	TCCACGAAGCC ATCATCCTCAT CA		63.78
*p21*	Forward	TGGCACCTCAC CTGCTCTG	179	61.89
*p21*	Reverse	GTTTGGAGTGG TAGAAATCTGT CAT		59.06
*GAPDH*	Forward	AATGGGCAGCC GTTAGGAAA	168	59.96
*GAPDH*	Reverse	GCGCCCAATAC GACCAAATC		59.97

### Western blotting (WB)

Identical quantities of protein (30 µg) were separated on a 10% SDS-PAGE gel and transferred onto nitrocellulose membranes. In the knockdown experiment, to ensure that even low levels of *F13B* could be accurately detected and quantified, we increased the sample loading amount. After blocking for an hour at room temperature with 5% nonfat milk in TBST buffer, the membranes were incubated overnight at 4 ^∘^C with primary antibodies against the target proteins. The primary antibodies used were *F13B* (cat No: 20269-1-AP), CD31 (cat No: 11265-1-AP), p-VEGFR2/VEGFR2 (cat No: 26415-1-AP), MMP2 (cat No: 10373-2-AP), MMP9 (cat No: 10375-2-AP), p-AKT/AKT (cat No: 28731-1-AP / 10176-2-AP), VEGF (cat No: 66828-1-Ig), Cyclin B1 (cat No: 55004-1-AP), CDC2 (cat No: 15646-1-AP), CDC25 (cat No: 55031-1-AP), p21 (cat No: 10355-1-AP) (1:1000), and Cyclin D1 (cat No: 60186-1-Ig), *VEGFA* (cat No: 19003-1-AP), p-mTOR/mTOR (cat No: 67778-1-Ig / 66888-1-Ig), HIF-1α (cat No: 20960-1-AP) (1:5000) (Wuhan Sanying Biotechnology, Wuhan, China). The membranes were then washed with TBST solution and incubated for an hour with HRP-conjugated secondary antibodies (1:5000, Abcam). Protein bands were detected using an enhanced chemiluminescence (ECL) kit (Beyotime) and imaged using the Bio-Rad ChemiDoc XRS+ System.

### Cell Counting Kit-8 (CCK-8)

5×10^3^ cells were seeded in each well of 96-well plates overnight. The CCK-8 test was used to assess cell viability at 1, 2, 3, 4, and 5 days post-transfection. After a second 2-h incubation at 37 ^∘^C, 10 µL of the CCK-8 solution was added to each well. Absorbance at 450 nm was measured using a microplate reader.

### Transwell assay

For the migration assay, serum-free media containing 2 × 10^4^ HCC cells per well were used in the top chamber of a 24-well Transwell plate, while medium with 10% FBS as a chemoattractant was placed in the lower chamber. The top chamber was coated with Matrigel (BD Biosciences) for the invasion experiment, and 5 × 10^4^ cells were seeded per well in serum-free media. After a 24-h incubation, non-invaded or non-migrated cells in the top chamber were removed with a cotton swab. Invaded or migrated cells on the membrane’s bottom were fixed with 4% paraformaldehyde and stained with DAPI. The stained cells were quantified using a microscope (Olympus, Tokyo, Japan).

### Flow cytometry

For flow cytometry analysis of the cell cycle, HCC cells were detached using trypsin-EDTA (Life Technologies Inc., Beijing, China) and washed with phosphate-buffered saline (PBS). The cells were then fixed overnight at 4 ^∘^C in 70% ethanol. After washing with PBS, cells were stained with propidium iodide (PI) in the presence of RNase A to quantify DNA content. A flow cytometer (Jiyuan, Guangzhou, China) was used to carry out the flow cytometry. Data were analyzed using FlowJo software (FlowJo, Hangzhou, China) to determine the cell distribution in the G1, S, and G2 phases.

### Enzyme-linked immunosorbent assay (ELISA)

The expression of proteins was measured using an ELISA. First, a capture antibody was applied to 96-well plates, which were left to sit at 4 ^∘^C overnight. The plates were blocked with 5% non-fat milk in PBS for 2 h. Samples were then incubated in the wells at 37 ^∘^C for 1 h. The detection antibody was added and incubated for 1 hour at 37 ^∘^C after washing with PBST. Following this, a secondary HRP-conjugated antibody was incubated at 37 ^∘^C for another hour. The substrate solution was added, and the reaction was stopped after 15 min with a stop solution. Finally, the absorbance at 450 nm was measured using a microplate reader (Thermo Scientific, Shanghai, China).

### Cytotoxicity assay

Lactate dehydrogenase (LDH) activity was assessed to evaluate cellular cytotoxicity. HUVECs were seeded in 96-well culture plates and treated with various concentrations of VEGF (0, 5, 10, 15, or 20 ng/mL) for 24 h. Cellular proteins were extracted and analyzed using a commercially available LDH activity assay kit from Jiangsu KeyGen BioTECH Corp., Ltd. (Nanjing, Jiangsu, China).

### Ethical approval and consent to participant

Not applicable.

### Statistical analysis

Statistical analysis was performed using the R programming language. All experiments were conducted in triplicate, and results are expressed as mean ± standard deviation (SD). One-way ANOVA was used to determine significance between multiple groups, with Tukey’s post-hoc test applied for pairwise comparisons. For analyses involving multiple variables, two-way ANOVA was used, with Bonferroni correction applied for multiple comparisons in post-hoc tests. A *P* value of < 0.05 was considered statistically significant.

## Results

### Classification and functional enrichment analysis of GSE120123-DEGs

**Figure 1.**
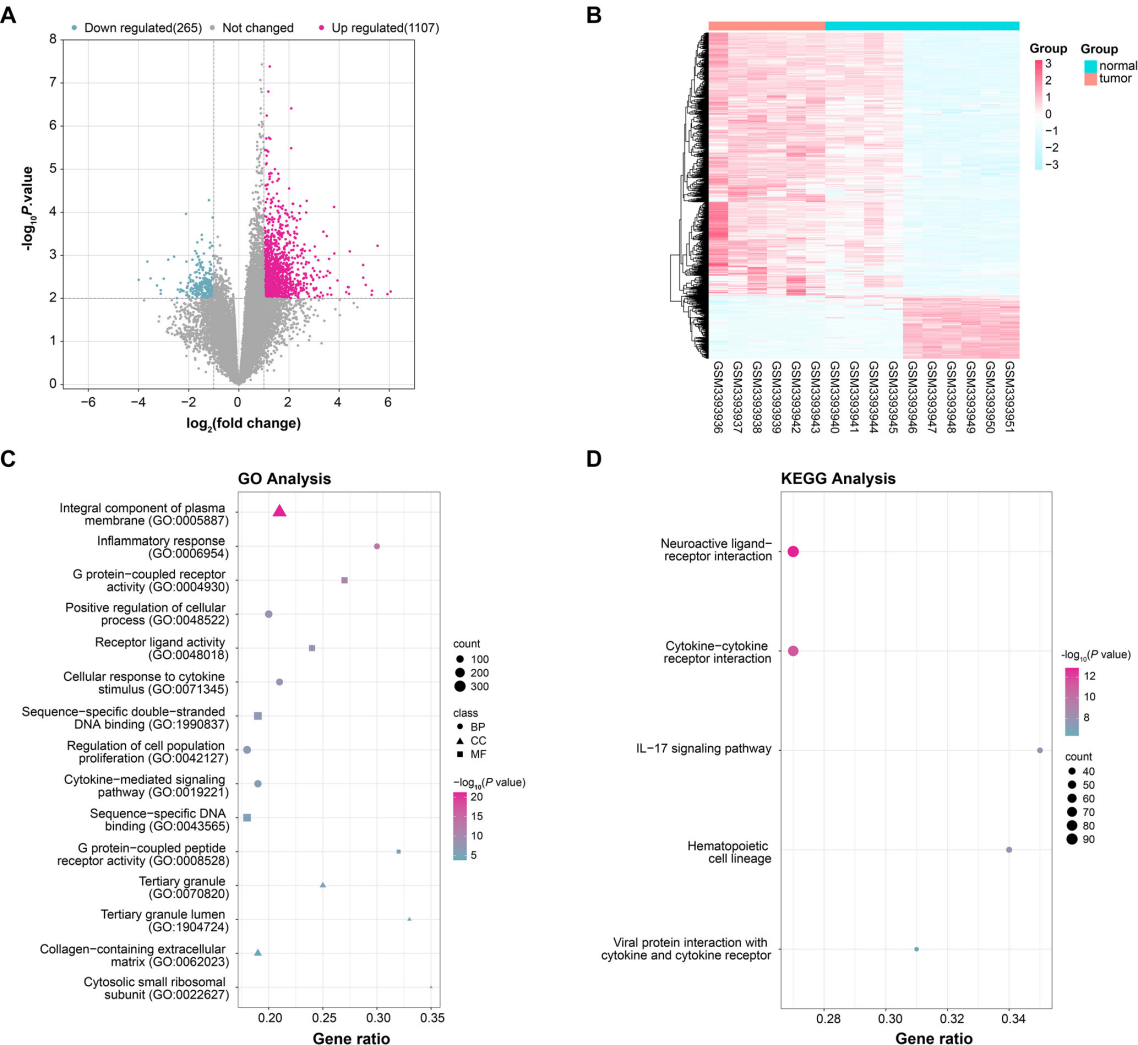
**Identification and functional enrichment analysis of DEGs in the GSE120123 dataset.** (A) Volcano plot showing the distribution of DEGs in the GSE120123 dataset. Red dots represent 1107 upregulated DEGs, blue dots represent 265 downregulated DEGs, and gray dots represent genes that did not meet the screening criteria; (B) Heatmap showing the expression of DEGs in the GSE120123 dataset. Pink columns represent tumor samples, while blue columns represent normal samples; (C) GO enrichment analysis of DEGs. Circles represent enrichment items in BP, triangles represent enrichment items in CC, and squares represent enrichment items in MF; (D) Bubble plot showing KEGG pathway enrichment analysis of DEGs. The size of the bubbles indicates the significance level of enrichment, the color intensity represents the enrichment fraction, and the horizontal axis indicates the gene ratio. DEGs: Differentially expressed genes; GO: Gene Ontology; BP: Biological process; CC: Cellular component; MF: Molecular function; KEGG: Kyoto Encyclopedia of Genes and Genomes.

From the GSE120123 dataset, 1372 DEGs were identified, including 1107 upregulated and 265 downregulated DEGs (Figure 1A). The cluster distribution of these DEGs in the dataset is shown in Figure 1B. The GO analysis revealed that these DEGs were enriched in terms like receptor ligand activity, cellular response to cytokine, and tertiary granule lumen (Figure 1C). KEGG pathway analysis showed significant enrichment in cytokine–cytokine receptor interaction, IL-17 signaling pathway, neuroactive ligand–receptor interaction, hematopoietic cell lineage, and viral protein interaction with cytokine and cytokine receptor pathways (Figure 1D).

### Screening and survival prognostic analysis of 220 overlapping genes

**Figure 2.**
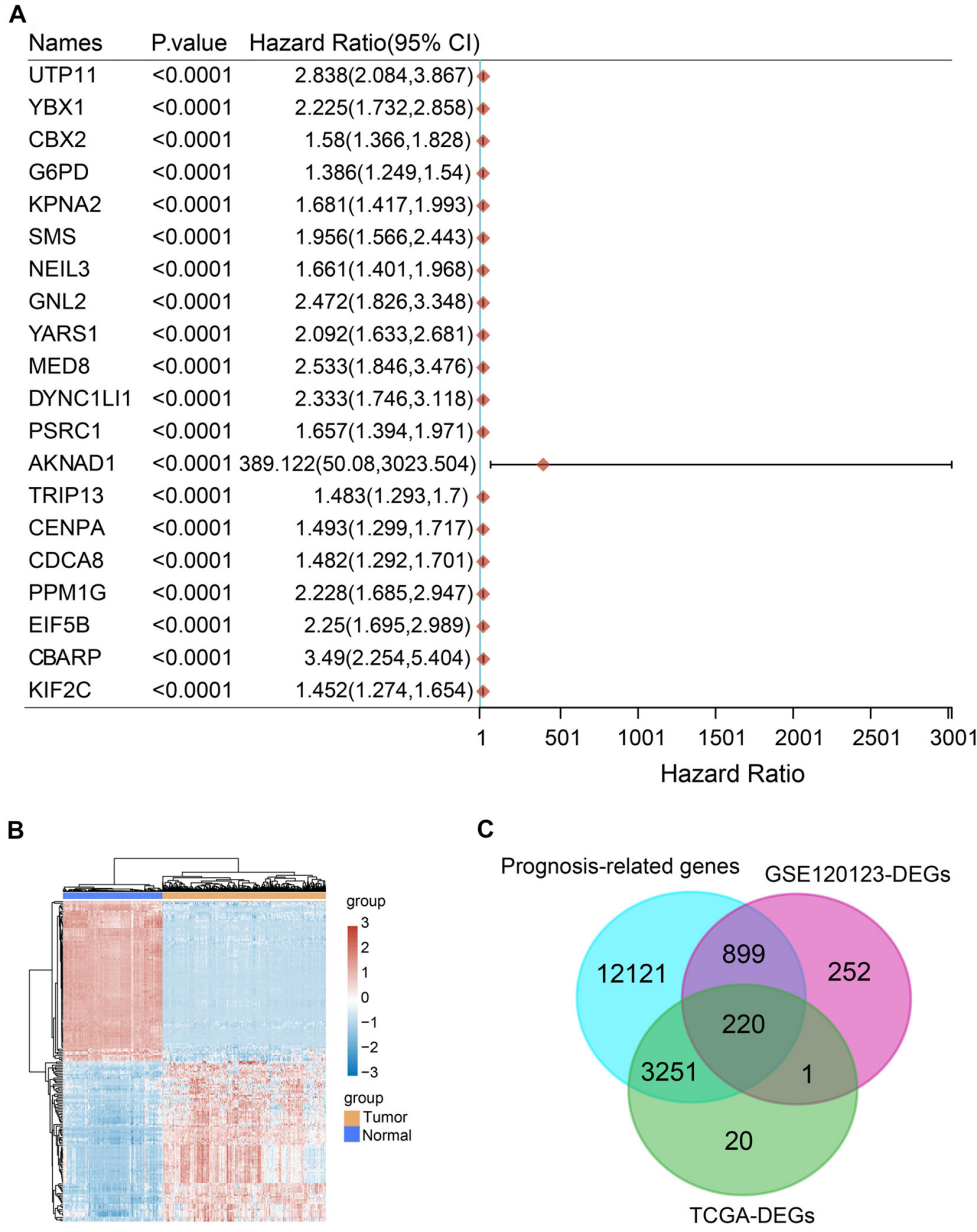
**Prognostic gene analysis in the TCGA-LIHC dataset.** (A) Univariate Cox analysis in the TCGA-LIHC dataset, presented as a forest plot showing the top 20 genes associated with LIHC prognosis; (B) Heatmap of DEGs based on the TCGA-LIHC database. Blue represents the control group, and yellow represents the tumor group; (C) Venn diagram showing the overlapping genes among the TCGA prognosis-related genes, GSE120123-DEGs, and TCGA-DEGs. TCGA: The Cancer Genome Atlas; LIHC: Liver hepatocellular carcinoma; DEGs: Differentially expressed genes.

**Figure S1.**
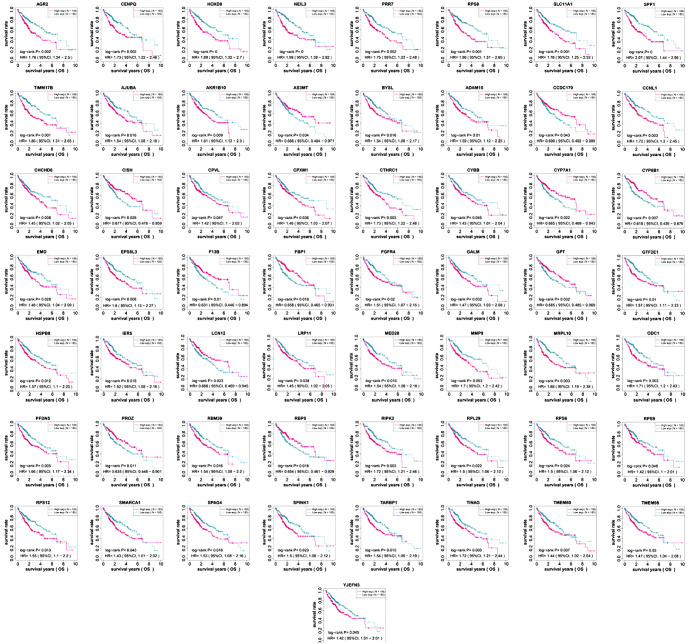
**OS analysis of 57 genes in LIHC.** KM survival curve showing OS analysis for 9 significant key genes (*P* < 0.05). The *x*-axis represents survival time, while the *y*-axis represents survival probability. The pink line represents high gene expression, while the blue line represents low expression. KM: Kaplan–Meier; OS: Overall survival.

In the TCGA-LIHC database, a comprehensive OS analysis of all genes identified 16,491 prognosis-related genes, with the top 20 highlighted in Figure 2A. Differential expression analysis of TCGA-LIHC samples identified 2558 upregulated and 934 downregulated DEGs (Figure 2B). Intersection analysis of prognostic genes, GSE120123-DEGs, and TCGA-LIHC-DEGs identified 220 overlapping genes for further investigation (Figure 2C). OS analysis on these genes revealed 57 with significant *P* values (Figure S1).

### LASSO-Cox regression analysis and prognostic model validation

**Figure S2.**
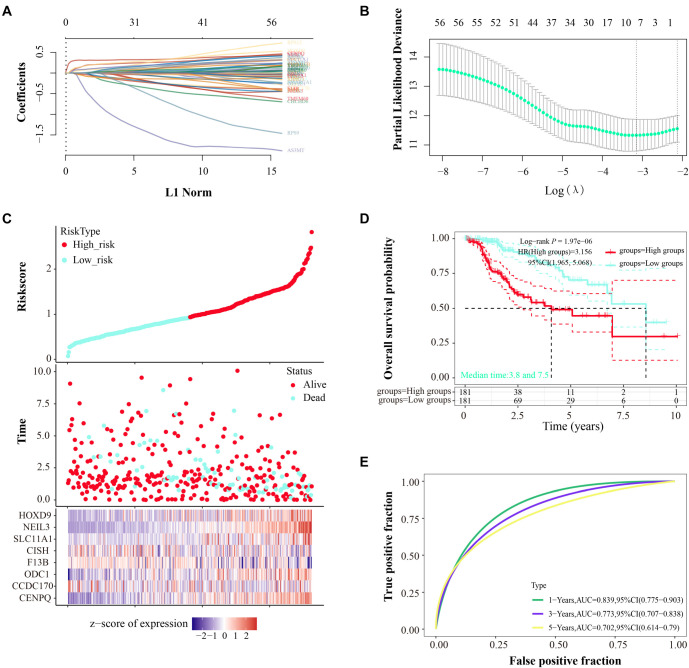
**Prognostic risk model for 57 key genes.** (A) Lasso coefficient map for 57 genes, each represented by a different colored line; (B) LASSO regression with ten-fold cross-validation identifying 8 prognostic genes based on the smallest λ value. Vertical dashed lines indicate regularization parameters chosen via cross-validation; (C) Risk model analysis: scatter plot depicting gene expression levels versus survival time/status in the TCGA dataset. Heat map shows the expression distribution of the eight prognostic genes in high- and low-risk groups; (D) KM survival analysis of high- and low-risk groups. The *x*-axis represents survival time in years, and the *y*-axis represents OS probability; (E) ROC curves for the prognostic model. The *x*-axis represents the false positive rate, and the *y*-axis represents the true positive rate. LASSO: Least Absolute Shrinkage and Selector Operation; TCGA: The Cancer Genome Atlas; KM: Kaplan–Meier; ROC: Receiver operating characteristic.

**Figure S3.**
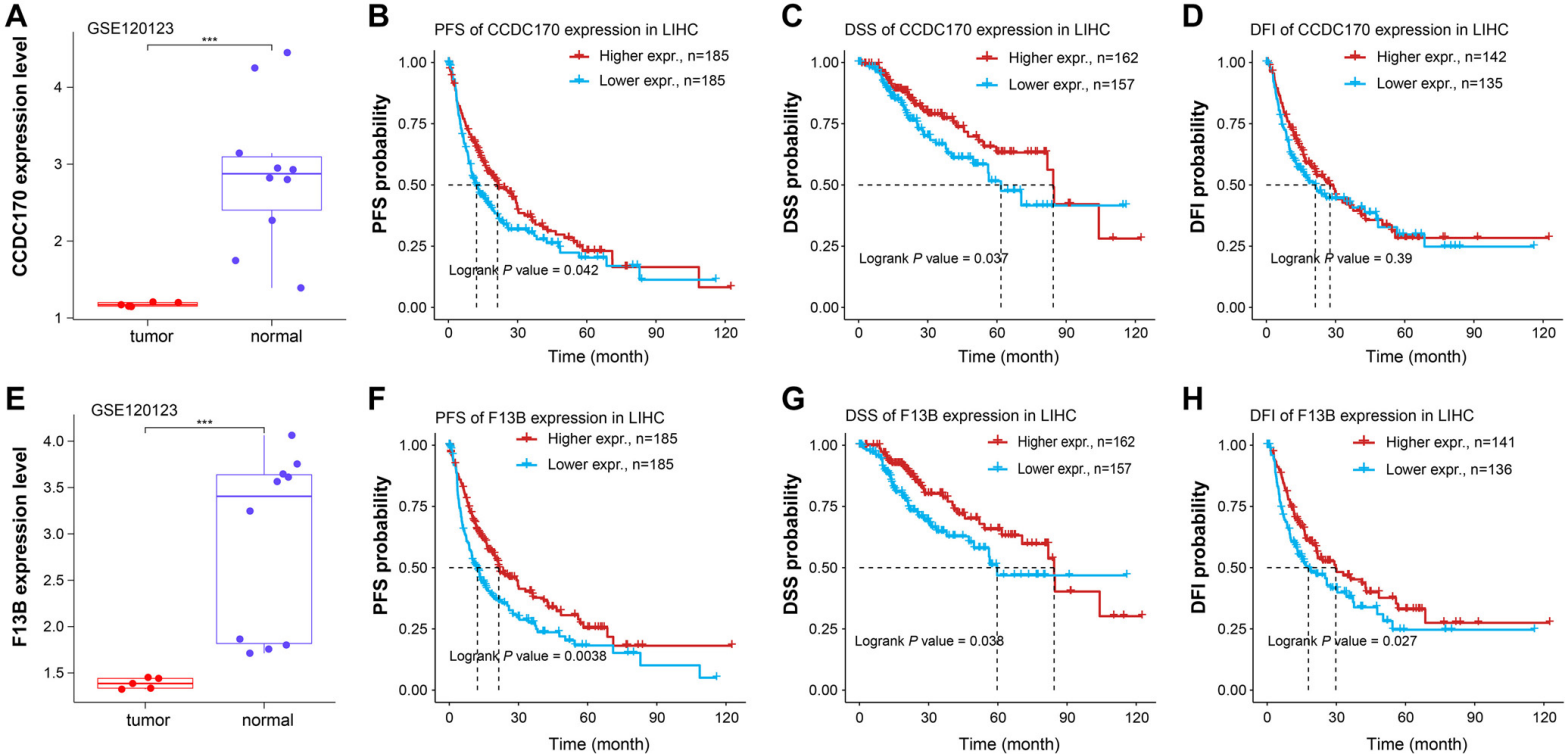
**Expression and survival analysis of CCDC170 and *F13B*.** (A) Comparison of CCDC170 expression in tumor and normal samples from the GSE120123 dataset. ***P < 0.001. (B–D) Relationship between CCDC170 expression and PFS, DSS, and DFI. Red lines indicate high expression, blue lines indicate low expression; (E) Comparison of *F13B* expression in tumor and normal samples in the GSE120123 dataset. ***P < 0.001. (F–H) Relationship between *F13B* expression and PFS, DSS, and DFI. Red lines indicate high expression, blue lines indicate low expression. DSS: Disease specific survival; PFS: Progression-free survival; DFI: Disease-free interval.

LASSO-Cox regression analysis was performed on the 57 genes with significant *P* values. Through ten-fold cross-validation, we identified eight key genes with λmin ═ 0.0429 (Figure S3A and S3B). The risk score formula for these genes was established as follows: Risk score ═ (0.1056) * CENPQ + (--0.0257) * CCDC170 + (0.044) * ODC1 + (--0.0212) * *F13B* + (--0.0043) * CISH + (0.0735) * SLC11A1 + (0.3065) * NEIL3 + (0.127) * HOXD9. TCGA-LIHC samples were divided into low-risk (*n* ═ 181) and high-risk (*n* ═ 181) groups based on gene expression. The risk score’s association with survival time/status for these genes is shown in Figure S2C. KM analysis indicated that OS probability was lower in high-risk patients compared to low-risk patients (Figure S2D). The predictive performance of the prognostic model was evaluated using ROC curve analysis. AUC values for predicting 1-, 3-, and 5-year outcomes were 0.839, 0.773, and 0.702, respectively, indicating significant prognostic value (Figure S2E).

**Figure 3.**
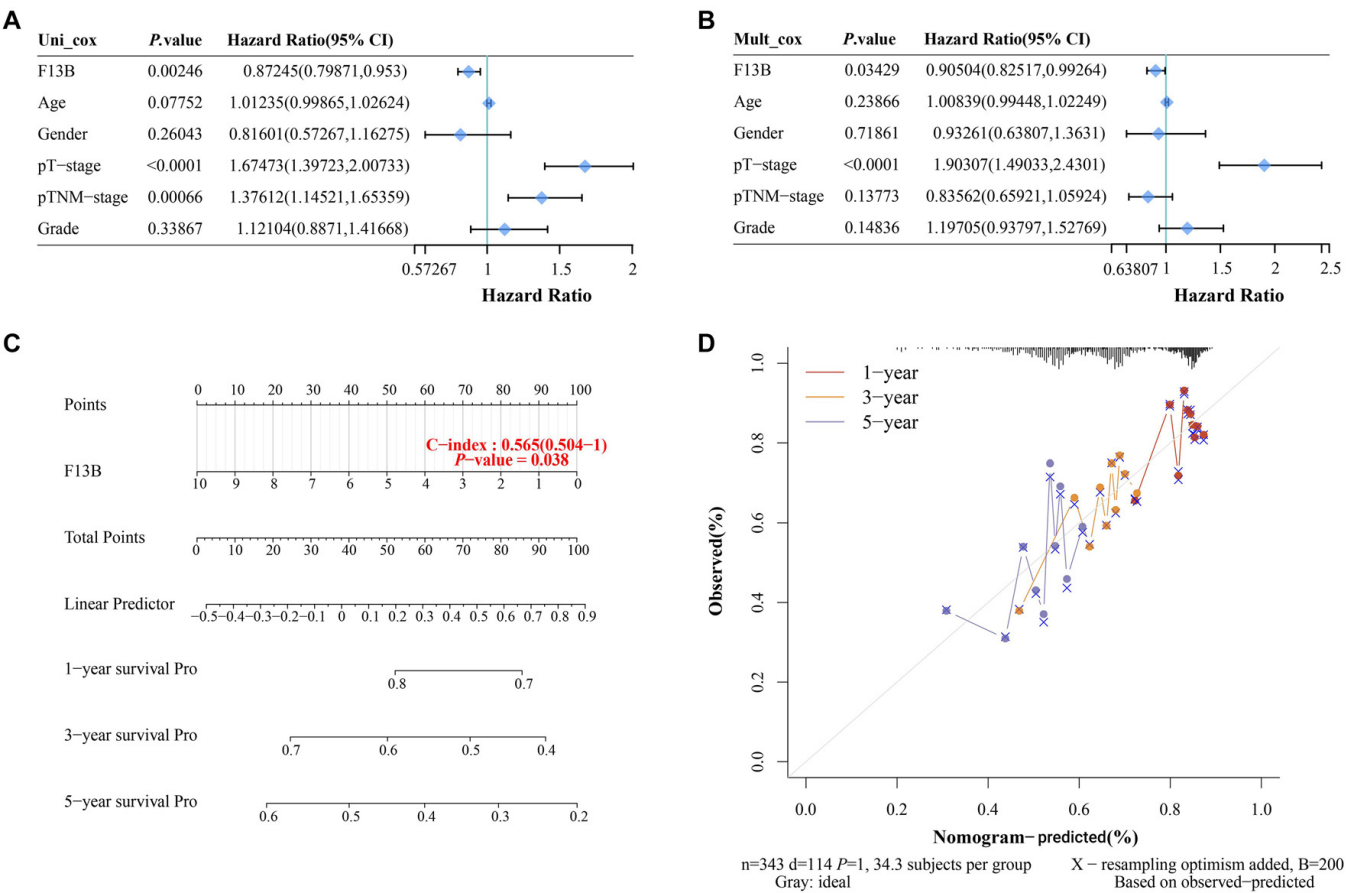
**Determination of independent prognostic parameters and construction of gene-based prognostic models.** (A and B) Forest plots of univariate and multivariate Cox proportional hazards regression analyses. HR and corresponding 95% CIs show the association between the independent variable *F13B* and OS, considering other clinical variables like age, sex, pT stage, pTNM stage, and grade. Each row represents a clinical variable, and horizontal lines represent null values (HR ═ 1). Points to the left of the line indicate a higher risk (HR > 1), while points to the right indicate a lower risk (HR < 1). (C) Nomogram predicting 1-, 3-, and 5-year OS in HCC patients based on *F13B*. The length of the line segment reflects the contribution of each variable to the final prediction. (D) Calibration plots of the nomogram for 1-, 3-, and 5-year OS predictions compared to actual outcomes. The *x*-axis represents predicted survival probabilities, while the *y*-axis represents observed probabilities. The diagonal line indicates perfect agreement. OS: Overall survival; HCC: Hepatocellular carcinoma.

### Expression analysis and prognosis assessment of key genes

Among the eight prognostic genes, CCDC170 and *F13B* had not been previously reported in HCC. We selected these two genes for further analysis. According to Figure S3A, CCDC170 expression was significantly lower in tumor samples than in normal samples (***P < 0.001). Survival analysis for CCDC170 showed that lower expression was linked to worse PFS (*P* ═ 0.042) and DSS (*P* ═ 0.037), but there was no significant correlation with DFI (*P* ═ 0.39) (Figure S3B–S3D). Similarly, *F13B* expression was lower in tumor samples compared to normal samples (Figure S3E). Lower *F13B* expression was associated with worse PFS (*P* ═ 0.0038), DSS (*P* ═ 0.038), and DFI (*P* ═ 0.027) (Figure S3F–S3H). These findings suggest that *F13B* is a significant prognostic factor in LIHC and warrants further investigation.

### Prognostic validation of *F13B* and nomogram construction for survival prediction

Univariate and multivariate Cox regression analyses validated the prognostic significance of *F13B*. In the univariate analysis (Figure 3A), *F13B* expression was strongly associated with OS (*P* ═ 0.00246, HR ═ 0.87245, 95% CI: 0.79781–0.953). Multivariate analysis confirmed that *F13B* was an independent prognostic factor for OS (*P* ═ 0.03429, HR ═ 0.90504, 95% CI: 0.82517–0.99264) (Figure 3B). A nomogram was constructed to predict 1-year, 3-year, and 5-year survival probabilities based on *F13B* expression (Figure 3C). The C-index for the nomogram was 0.565 (95% CI: 0.504–1), and the *P* value was 0.038, indicating moderate predictive accuracy. Calibration curves showed a good alignment between predicted and observed survival probabilities at one, three, and five years (Figure 3D). These results underscore the prognostic value of *F13B* in HCC, supporting its role as a reliable biomarker for predicting patient outcomes.

**Figure 4.**
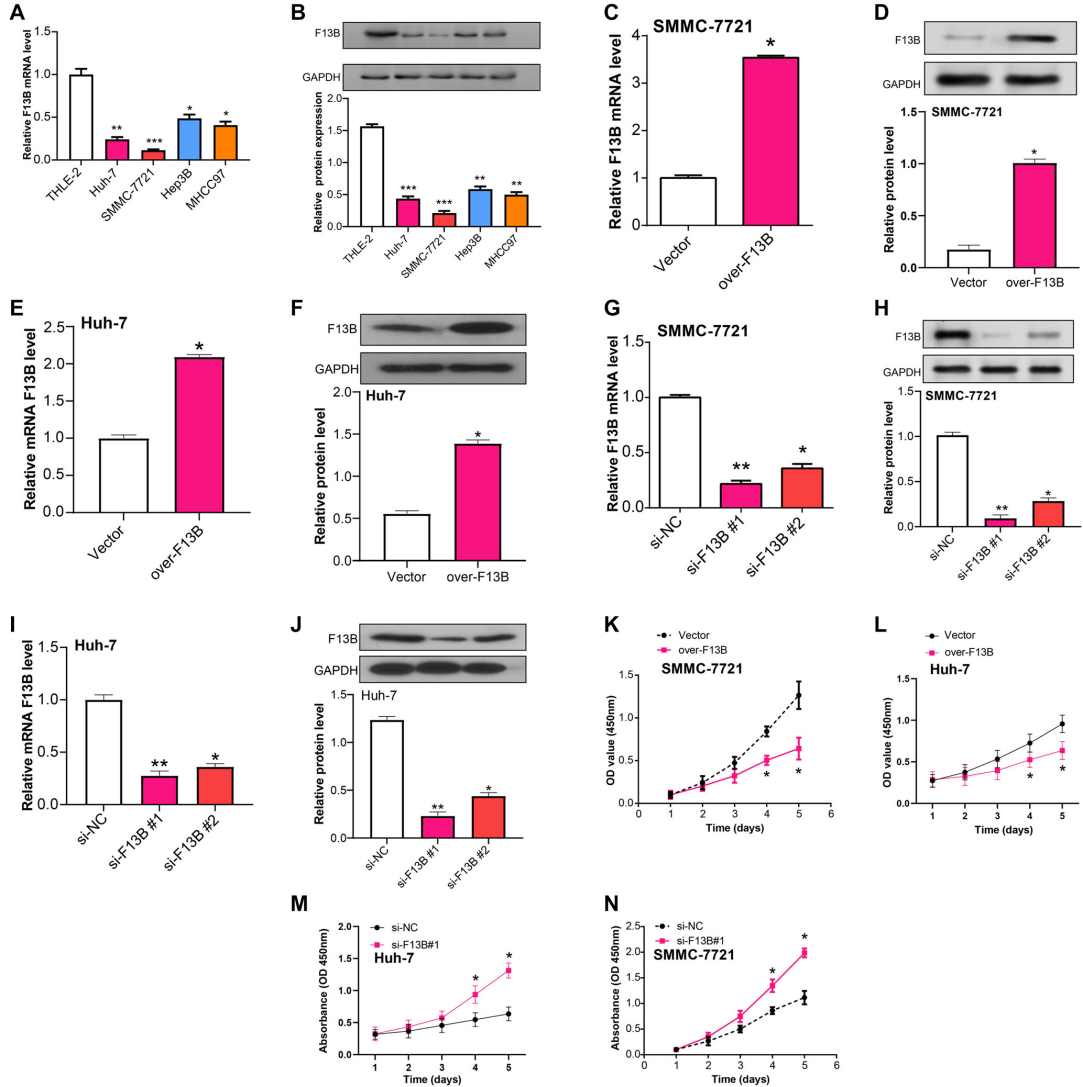
***F13B* knockdown enhances the proliferation of HCC cells.** (A and B) Relative *F13B* mRNA and protein levels in the normal liver cell line THLE-2 and HCC cell lines (Huh-7, SMMC-7721, Hep3B, MHCC97) measured via qRT-PCR and WB assays; (C and D) qRT-PCR and WB analysis of *F13B* mRNA and protein levels in SMMC-7721 cells transfected with either a control vector or overexpressing *F13B*; (E and F) qRT-PCR and WB analysis of *F13B* mRNA and protein levels in Huh-7 cells transfected with either a control vector or overexpressing *F13B*; (G and H) qRT-PCR and WB analysis of *F13B* mRNA and protein levels in SMMC-7721 cells transfected with si-NC or two different *F13B* siRNAs (si-*F13B*#1, si-*F13B*#2); (I and J) qRT-PCR and WB analysis of *F13B* mRNA and protein levels in Huh-7 cells transfected with si-NC or two different *F13B* siRNAs (si-*F13B*#1, si-*F13B*#2); (K and L) Cell proliferation analysis of SMMC-7721 and Huh-7 cells transfected with control or *F13B* overexpression, determined by CCK-8 assay; (M and N) Cell proliferation analysis of Huh-7 and SMMC-7721 cells transfected with si-NC or si-*F13B*#1, determined by CCK-8 assay. *P < 0.05, **P < 0.01. HCC: Hepatocellular carcinoma; qRT-PCR: Quantitative real-time polymerase chain reaction; WB: Western blotting; CCK-8: Cell Counting Kit-8.

**Figure 5.**
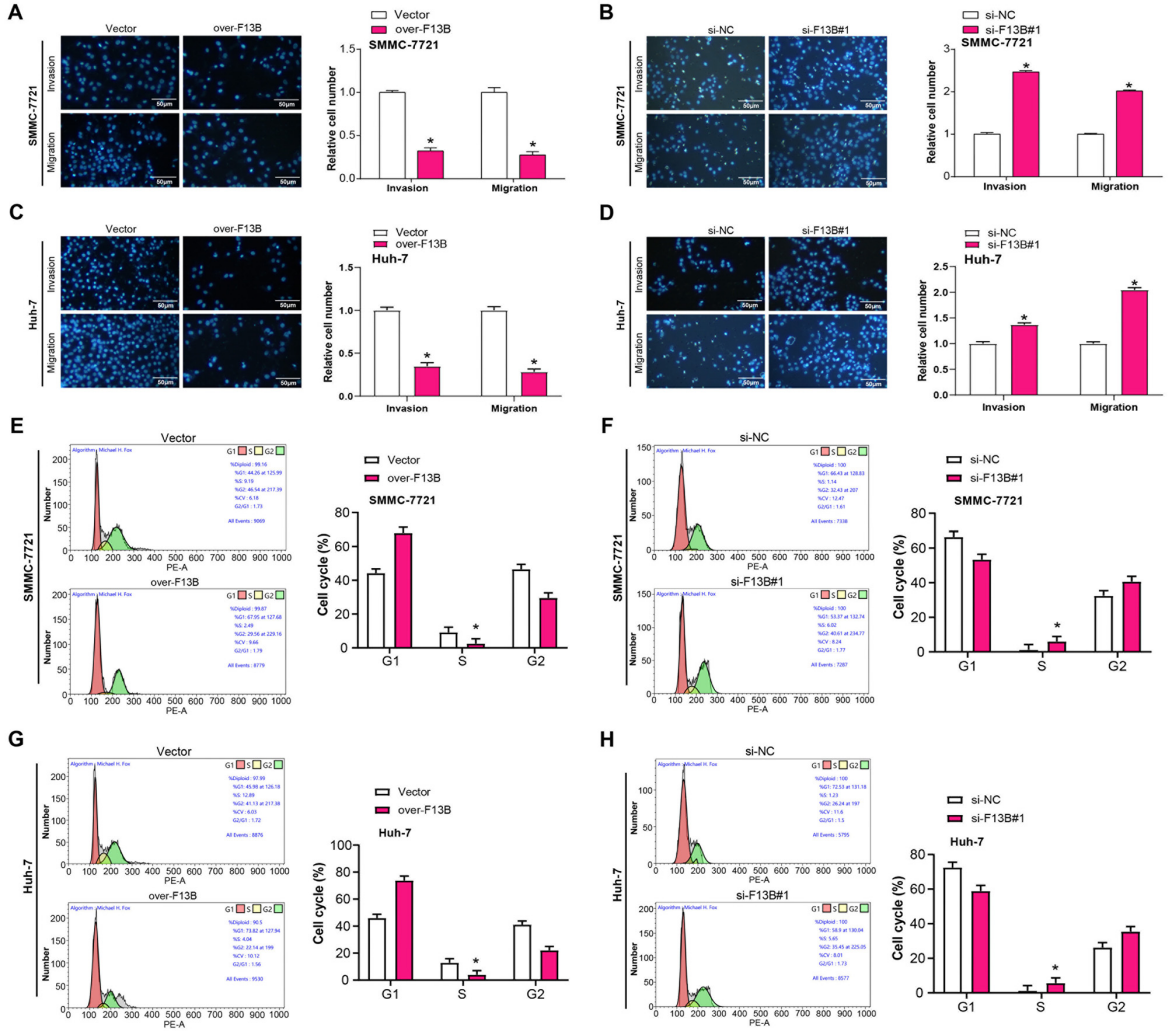
**Functional effects of *F13B* on cell invasion, migration, and cell cycle progression.** (A–D) Transwell assay results showing the effect of *F13B* overexpression (over-*F13B*) or knockdown (si-*F13B*#1) on the invasion and migration of SMMC-7721 and Huh-7 cells. Bar graphs quantify invaded and migrated cells; (E–H) Flow cytometry analysis of the cell cycle distribution in SMMC-7721 and Huh-7 cells after *F13B* overexpression or knockdown. Bar graphs show the percentage of cells in the G1, S, and G2 phases. *P < 0.05. Scale bar: 50 µm.

### Correlation of *F13B* expression with clinical characteristics in LIHC

**Figure S4.**
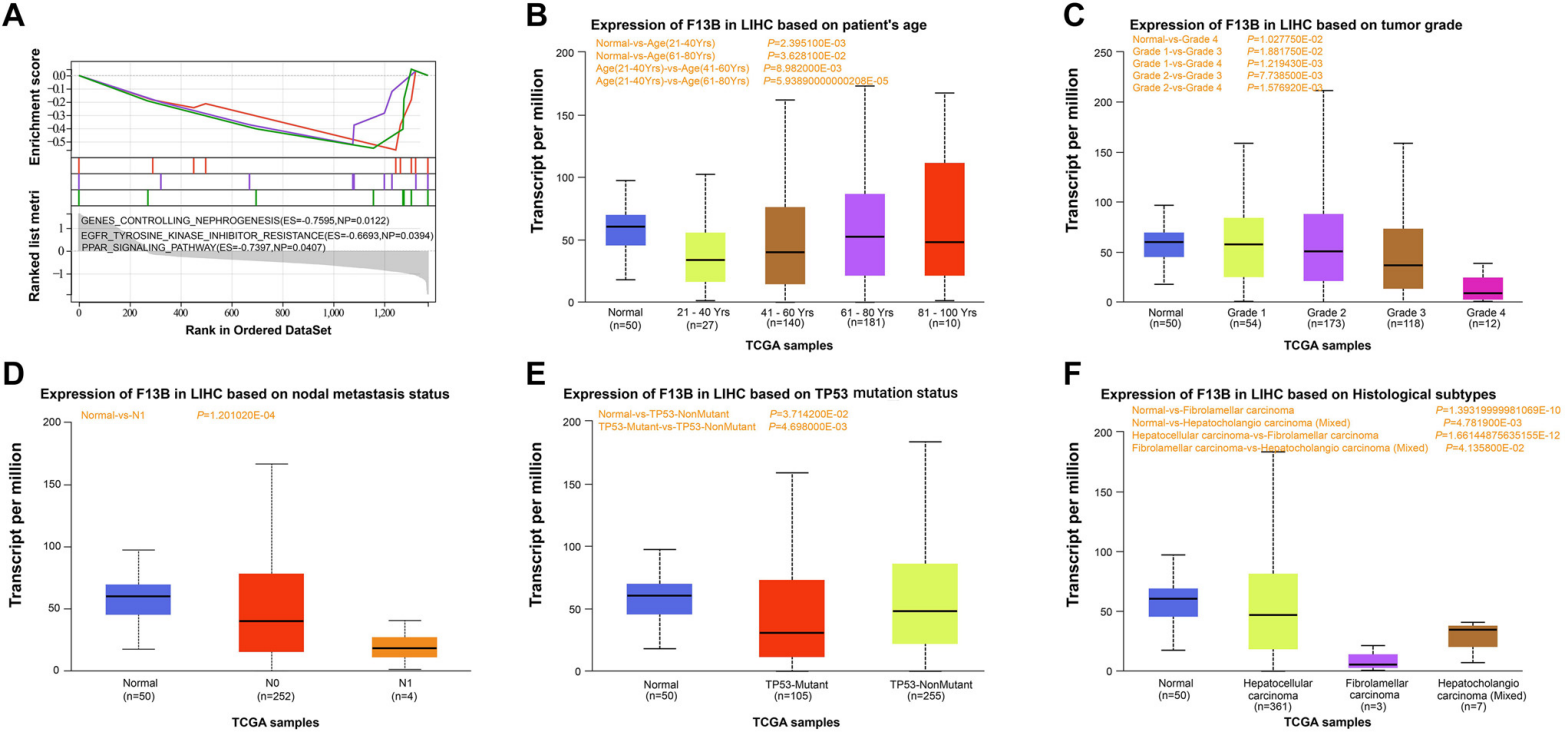
**Correlation of *F13B* expression with clinical features.** (A) *F13B*-related GSEA pathway. ES and NP are used to evaluate gene set enrichment significance; (B–F) *F13B* expression in LIHC based on age, tumor grade, nodal metastasis status, TP53 mutation status, and histological subtypes. GSEA: Gene set enrichment analysis; ES: Enrichment score; NP: Nominal *P* value; LIHC: Liver hepatocellular carcinoma.

GSEA-enriched pathways for *F13B* included genes controlling nephrogenesis, the PPAR signaling pathway, and EGFR tyrosine kinase inhibitor resistance (Figure S4A). *F13B* mRNA expression was analyzed across different clinical subgroups based on patient age, TP53 mutation status, nodal metastasis status, tumor grade, and histological subtypes. *F13B* expression increased with patient age (Figure S4B), decreased with higher tumor grades (Figure S4C), and was higher in the N0 nodal metastasis group (Figure S4D). TP53-NonMutant patients exhibited higher *F13B* expression than TP53-Mutant patients (Figure S4E). Among different histological subtypes, *F13B* expression was highest in HCC (Figure S4F).

### *F13B* regulates the proliferation, invasion, migration, and cell cycle of HCC cells

In cell line experiments, *F13B* expression levels were found to be significantly reduced in HCC cell lines, particularly in SMMC-7721 and Huh-7 (Figure 4A and 4B). The knockdown and overexpression efficiency of *F13B* in SMMC-7721 and Huh-7 cells was confirmed, with si-*F13B*#1 showing the most significant knockdown efficiency (Figure 4C–4J). CCK-8 assays revealed that *F13B* overexpression inhibited SMMC-7721 cell proliferation, while knockdown enhanced proliferative capacity (Figure 4K–4N). Transwell assays showed that *F13B* overexpression impeded SMMC-7721 cell migration and invasion, while knockdown promoted these processes (Figure 5A–5D). Flow cytometry analysis indicated that *F13B* overexpression reduced the proportion of cells in the S phase and increased the proportion in the G1 phase, while *F13B* knockdown caused the opposite effect (Figure 5E–5H).

**Figure 6.**
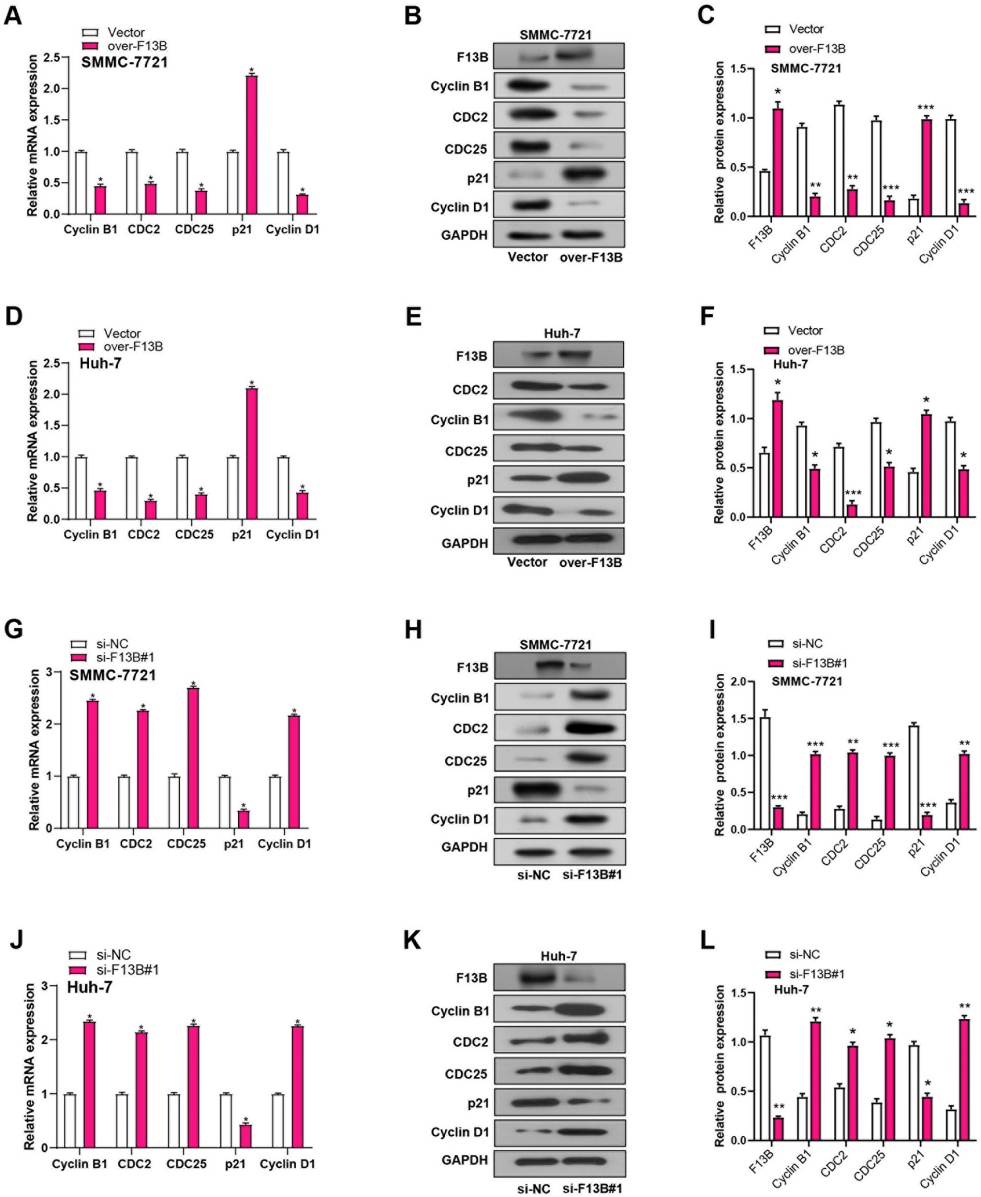
**Effects of knockdown or overexpression of *F13B* on cyclin expression in HCC.** (A–F) After *F13B* overexpression, changes in cyclin expression (Cyclin B1, CDC2, CDC25, p21, and Cyclin D1) in HCC cells were measured via qRT-PCR and WB; (G–L) After *F13B* knockdown, changes in cyclin expression in HCC cells were measured via qRT-PCR and WB. *P < 0.05. HCC: Hepatocellular carcinoma; qRT-PCR: Quantitative real-time polymerase chain reaction; WB: Western blotting.

To investigate the molecular mechanisms by which *F13B* regulates the cell cycle, we conducted qRT-PCR and WB assays to assess the expression levels of key cell cycle-related proteins, including CDC2, Cyclin B1, CDC25, p21, and Cyclin D1, in SMMC-7721 and Huh-7 cells. Overexpression of *F13B* significantly reduced the expression levels of Cyclin B1, CDC2, CDC25, and Cyclin D1 while increasing p21 levels (Figure 6A–6F). Conversely, *F13B* knockdown had the opposite effects, increasing Cyclin B1, CDC2, CDC25, and Cyclin D1 while reducing p21 expression (Figure 6G–6L). These findings suggest that *F13B* plays a crucial role in regulating cell cycle progression, likely through the modulation of these key proteins.

### Knockdown of *F13B* promoted the proliferation and migration of HUVEC

**Figure 7.**
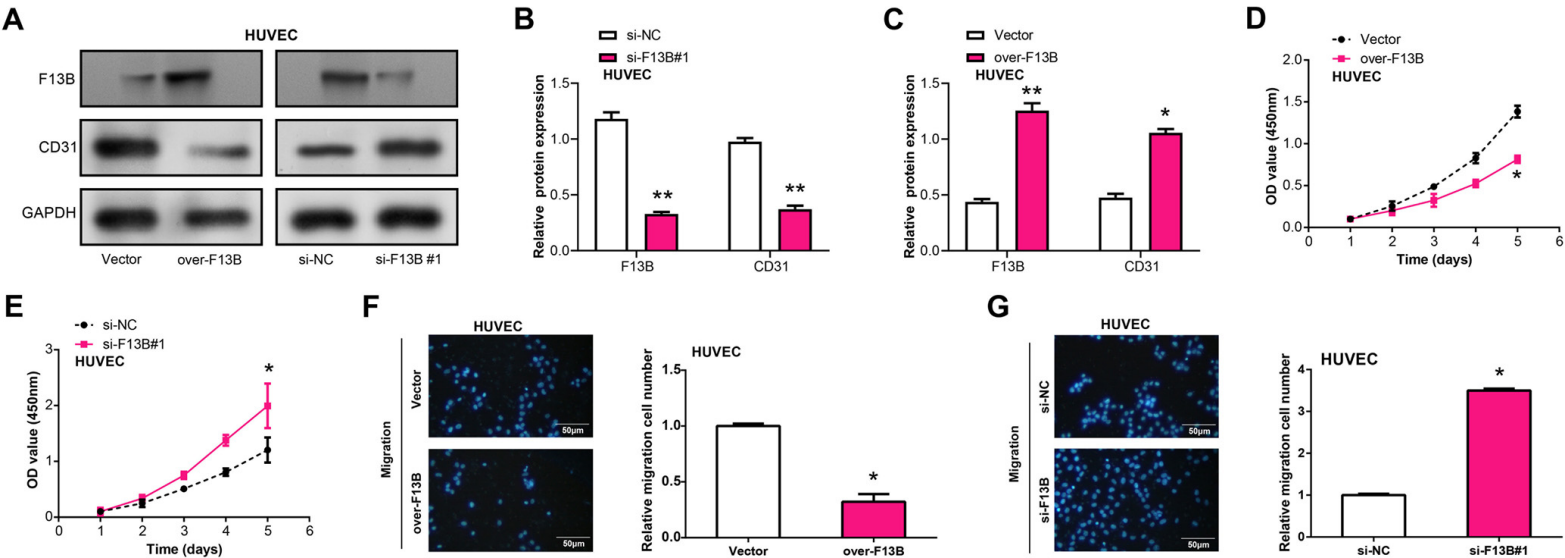
**Knockdown of *F13B* enhances expression of microvascular marker CD31 and promotes proliferation and migration of HUVECs.** (A–C) WB analysis of CD31 protein levels in HUVECs transfected with a vector, overexpressing *F13B*, si-NC, or si-*F13B*#1. GAPDH was used as the loading control; (D and E) CCK-8 assay showing the effect of *F13B* knockdown or overexpression on HUVEC proliferation; (F and G) Transwell assay showing the effect of *F13B* knockdown or overexpression on HUVEC migration. *P < 0.05. Scale bar: 50 µm. HUVEC: Human umbilical vein endothelial cells; WB: Western blotting; CCK-8: Cell Counting Kit-8; HCC: Hepatocellular carcinoma.

HUVEC is commonly used as a model for studying angiogenesis [25]. In this study, we analyzed the effects of *F13B* knockdown or overexpression on the expression of the microvascular marker CD31 in HUVEC via Western blot (WB) experiments. Our results demonstrated that *F13B* knockdown led to an upregulation of CD31 protein levels, while overexpression of *F13B* caused a decrease in CD31 expression (Figure 7A–7C). Functional assays further revealed that *F13B* overexpression inhibited HUVEC proliferation and migration, while knockdown of *F13B* had the opposite effect (Figure 7D–7G). These findings suggest that *F13B* plays a crucial role in regulating HUVEC angiogenesis.

### Overexpression of *F13B* inhibits VEGF-induced proliferation and invasion of HUVEC

**Figure 8.**
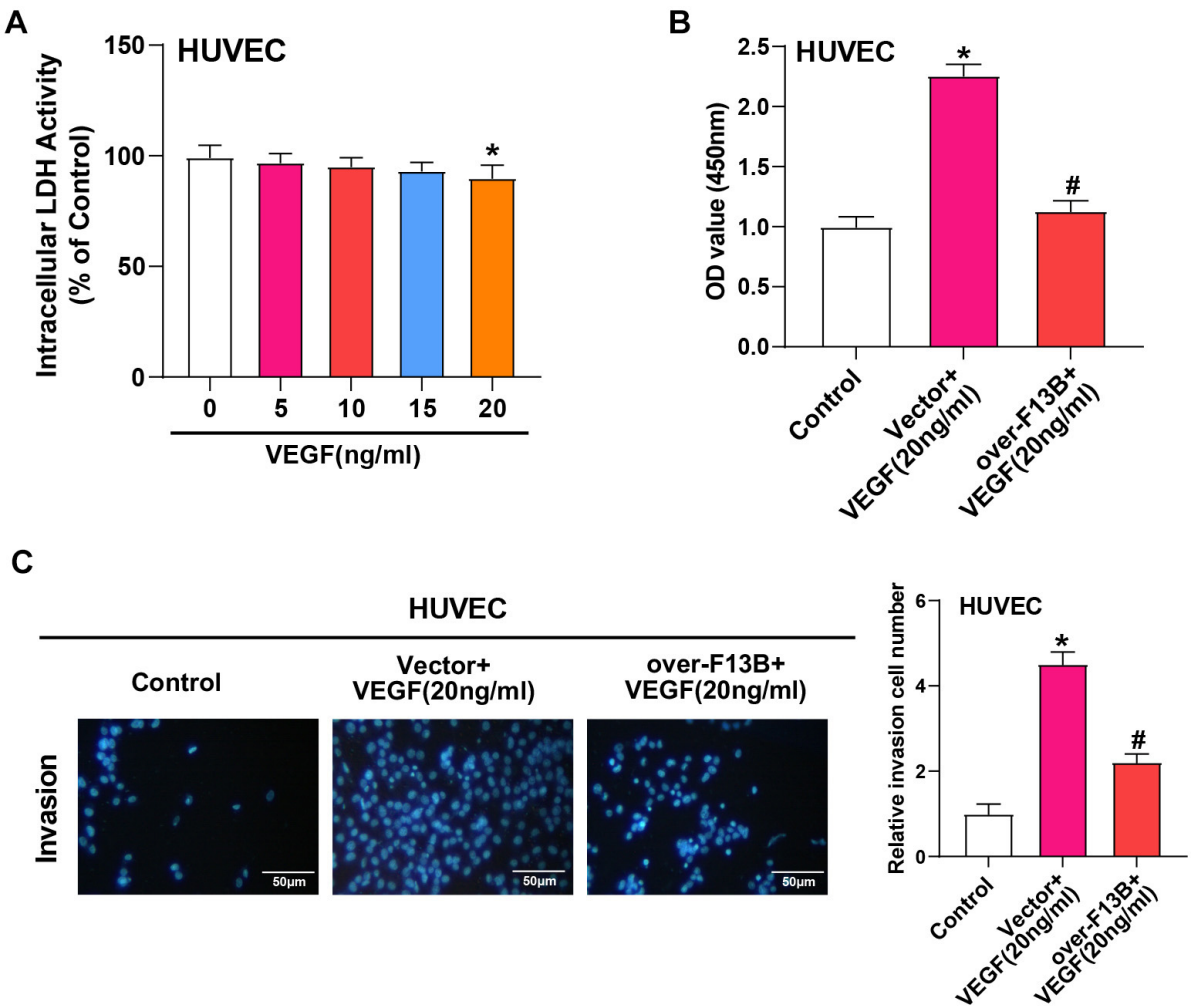
**Overexpression of *F13B* inhibits VEGF-induced HUVEC proliferation and invasion.** (A) LDH detection kit measuring the effects of different VEGF concentrations (0, 5, 10, 15, 20 ng/mL) on HUVEC toxicity after 24 h; (B) CCK-8 assay showing the proliferation of HUVECs after *F13B* overexpression with or without VEGF (20 ng/mL) treatment for 24 h; (C) Transwell assay showing HUVEC invasion after *F13B* overexpression with or without VEGF (20 ng/mL) treatment for 24 h. *P < 0.05 vs control, ^#^*P* < 0.05 vs vector + VEGF (20 ng/mL). Scale bar: 50 µm. LDH: Lactate dehydrogenase; HUVEC: Human umbilical vein endothelial cells; CCK-8: Cell Counting Kit-8; VEGF: Vascular endothelial growth factor.

The LDH activity assay is commonly used to evaluate cell membrane integrity and cytotoxicity [26]. When cell membranes are compromised, LDH is released into the culture medium, and an increase in LDH activity is indicative of cytotoxicity. Using an LDH detection kit, we assessed the impact of different concentrations of VEGF (0, 5, 10, 15, 20 ng/mL) on HUVEC cytotoxicity after 24 h. A significant decrease in cytotoxicity was observed only at 20 ng/mL VEGF (Figure 8A). Additionally, CCK-8 and transwell assays showed that 20 ng/mL VEGF significantly increased the viability and invasive capacity of HUVEC cells, but this increase was attenuated by *F13B* overexpression (Figure 8B and 8C).

### *F13B* inhibits HUVEC proliferation by downregulating the expression of *VEGFA*

**Figure 9.**
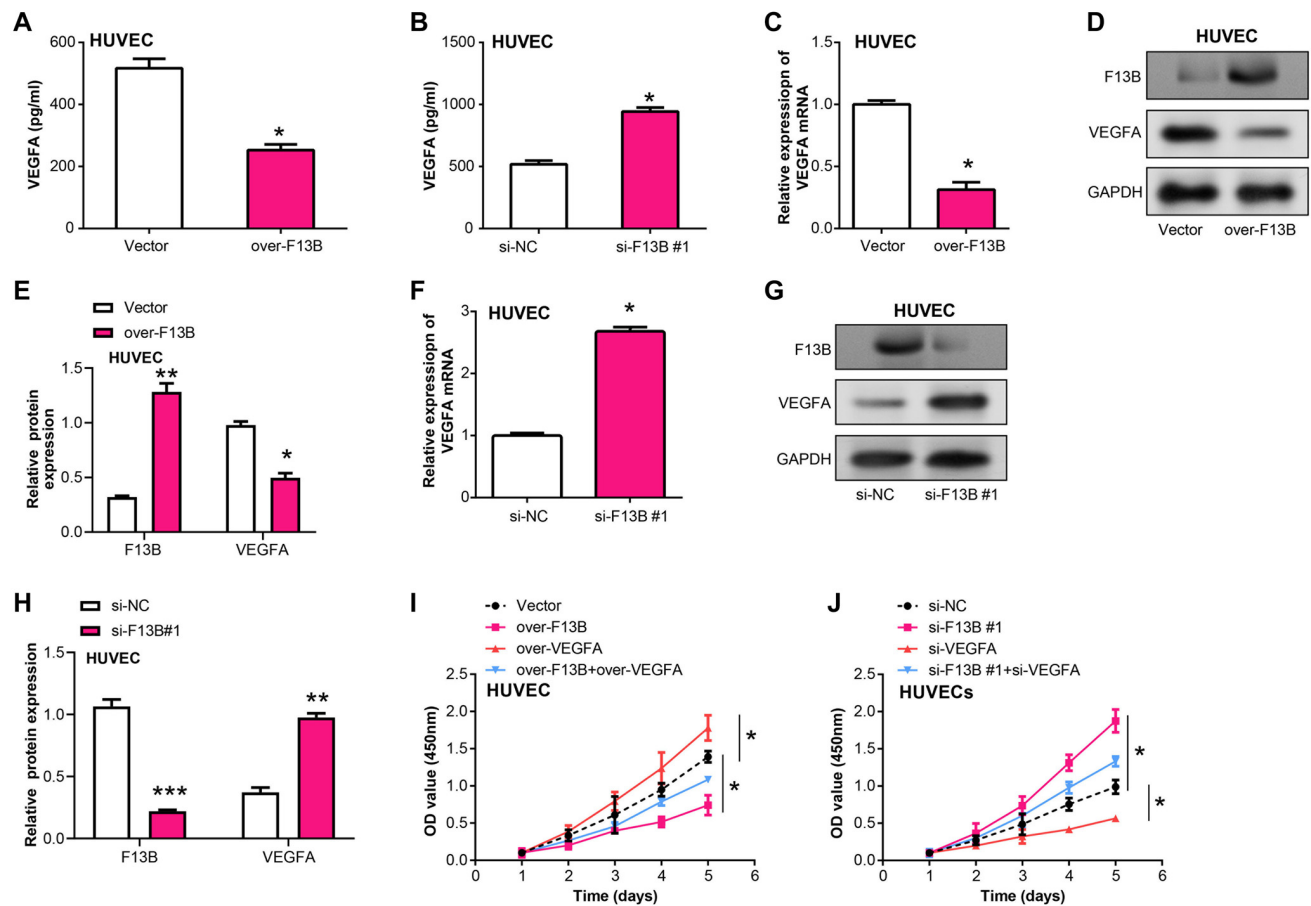
***F13B* regulates *VEGFA* expression and affects proliferation of HUVEC cells.** (A) ELISA showing *VEGFA* secretion in the supernatant of HCC cells overexpressing *F13B*; (B) ELISA showing *VEGFA* secretion in the supernatant of HCC cells with *F13B* knockdown; (C–E) qRT-PCR and WB examining *VEGFA* mRNA/protein expression in HCC cells after *F13B* overexpression; (F–H) qRT-PCR and WB examining *VEGFA* mRNA/protein expression in HCC cells after *F13B* knockdown; (I and J) CCK-8 assay detecting HUVEC proliferation after overexpression of *F13B* and *VEGFA*, or *F13B* knockdown and *VEGFA* knockdown. *P < 0.05. HUVEC: Human umbilical vein endothelial cells; ELISA: Enzyme-linked immunosorbent assay; HCC: Hepatocellular carcinoma; qRT-PCR: Quantitative real-time polymerase chain reaction; WB: Western blotting; CCK-8: Cell Counting Kit-8.

*VEGFA* is a major angiogenic factor critical in tumor angiogenesis [27]. We examined the regulation of *VEGFA* by *F13B* in HUVEC cells. ELISA results indicated that *F13B* overexpression suppressed *VEGFA* secretion in the HUVEC supernatant (Figure 9A), while *F13B* knockdown promoted it (Figure 9B). Subsequent qRT-PCR and WB experiments confirmed that *F13B* negatively regulates *VEGFA* expression at both the mRNA and protein levels (Figures 9C--9H). *F13B* overexpression also inhibited HUVEC proliferation, while overexpression of *VEGFA* rescued this inhibition (Figure 9I). Conversely, the knockdown of *F13B* stimulated HUVEC growth, but this effect was reversed by *VEGFA* knockdown (Figure 9J).

### *F13B* regulates key angiogenesis markers and AKT/mTOR signaling pathway in VEGF-treated HUVEC

**Figure 10.**
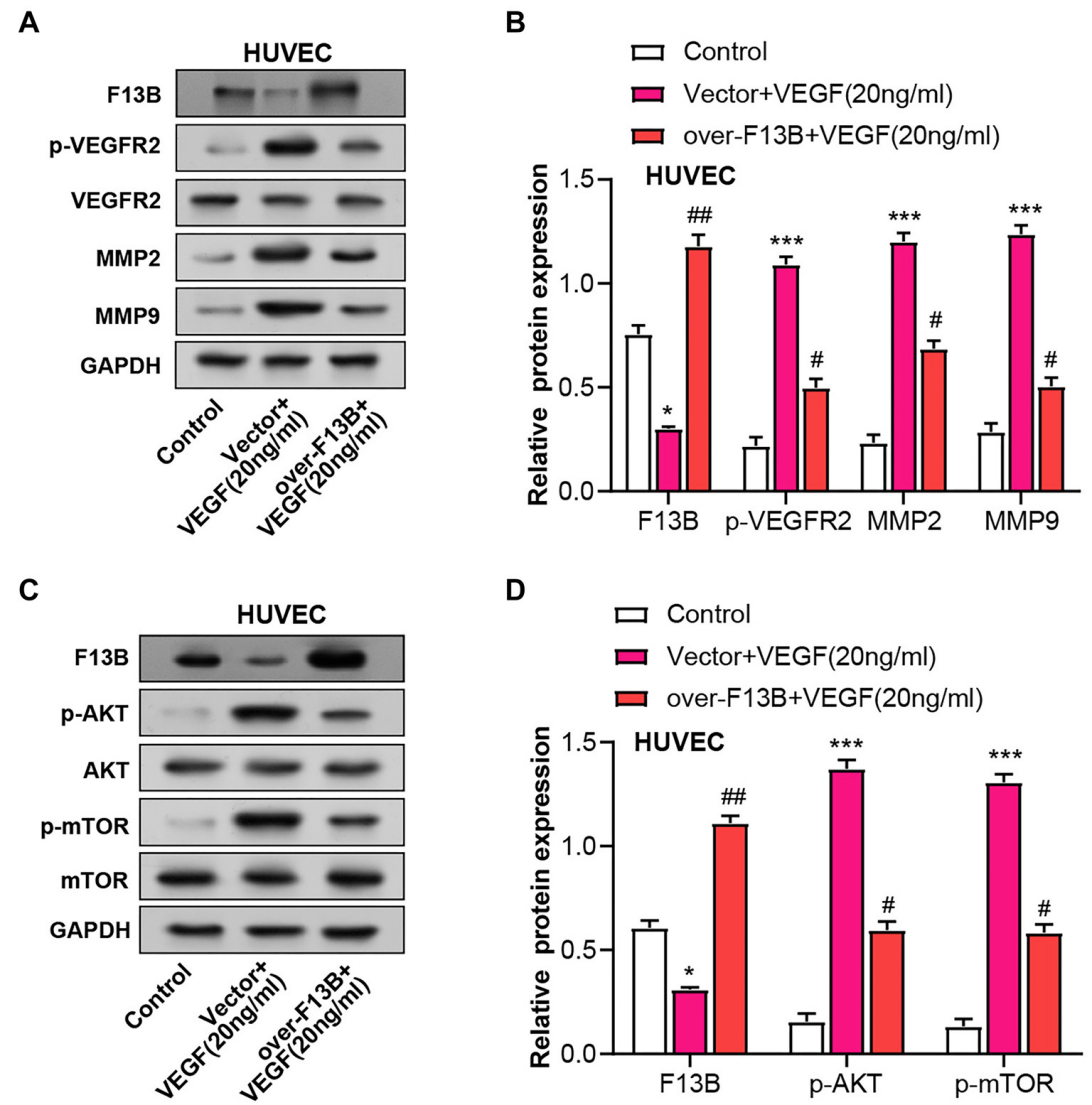
***F13B* regulates key angiogenesis markers and AKT/mTOR signaling in HUVECs.** (A–D) WB detecting changes in the expression of p-VEGFR2, VEGFR2, MMP2, and MMP9 in HUVECs overexpressing *F13B* with or without VEGF (20 ng/mL) treatment for 24 h; (E–G) WB detecting p-AKT, AKT, p-mTOR, and mTOR expression in HUVECs overexpressing *F13B* with or without VEGF (20 ng/mL) treatment for 24 h. HUVEC: Human umbilical vein endothelial cells; WB: Western blotting.

VEGF treatment (20 ng/mL) significantly increased the levels of p-VEGFR2, MMP9, and MMP2 in HUVEC, though it did not affect VEGFR2 levels (Figure 10A–10D). Overexpression of *F13B* reduced the levels of these proteins. Additionally, WB analysis showed that VEGF treatment raised p-mTOR and p-AKT protein levels in HUVEC, but *F13B* overexpression alleviated this increase (Figure 10E–10G). These findings indicate that *F13B* suppresses angiogenesis by inhibiting the AKT/mTOR signaling pathway and downregulating VEGFR2, MMP2, and MMP9.

### *F13B* affects HCC through the HIF-1α/VEGF pathway

**Figure 11.**
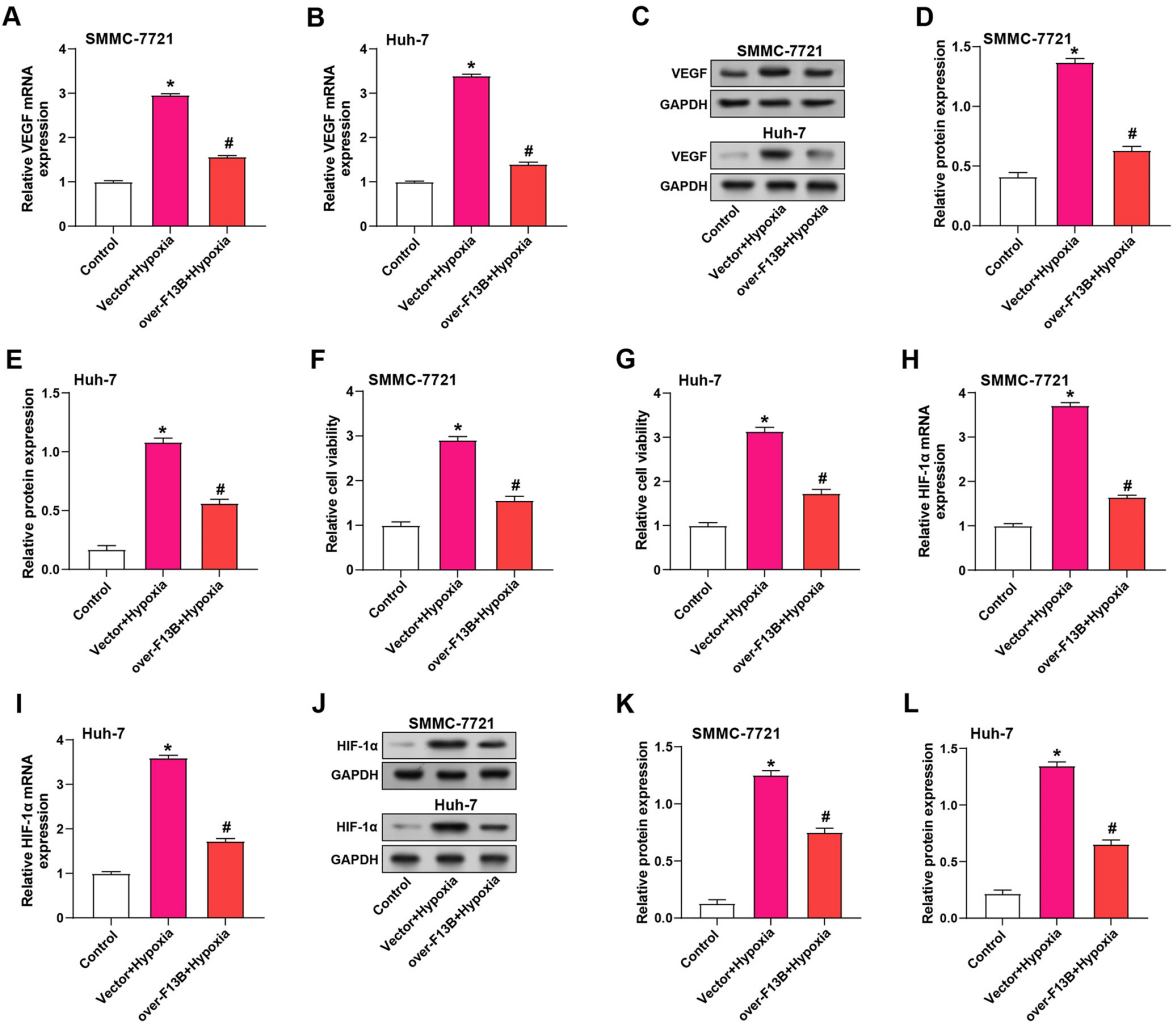
***F13B* affects HCC through the HIF-1α/VEGF pathway.** (A–E) WB detecting VEGF expression in SMMC-7721 and Huh-7 cells after *F13B* overexpression with or without hypoxic conditions; (F and G) CCK-8 assay showing SMMC-7721 and Huh-7 cell viability after *F13B* overexpression with or without hypoxia; (H–L) qRT-PCR and WB detecting HIF-1α expression in SMMC-7721 and Huh-7 cells after *F13B* overexpression with or without hypoxia. *P < 0.05 vs control, ^#^*P* < 0.05 vs vector + Hypoxia. HCC: Hepatocellular carcinoma; qRT-PCR: Quantitative real-time polymerase chain reaction; WB: Western blot; CCK-8: Cell Counting Kit-8.

We also examined the effect of *F13B* on VEGF expression in SMMC-7721 and Huh-7 cells. qRT-PCR and WB results showed that hypoxic conditions significantly increased VEGF expression, which was reduced by *F13B* overexpression (Figure 11A–11E). Under hypoxic conditions, cell viability increased, but this effect was mitigated by *F13B* overexpression (Figure 11F and 11G). *HIF1A*, a key regulator of the cellular response to low oxygen, is known to upregulate VEGF expression. Our analyses revealed that *F13B* overexpression also downregulated HIF-1α levels in hypoxic conditions (Figure 11H–11L), suggesting that *F13B* modulates HCC progression through the HIF-1α/VEGF pathway.

## Discussion

HCC is one of the main causes of cancer-related deaths worldwide, characterized by its aggressive nature and poor prognosis [28]. Understanding the molecular mechanisms underlying HCC is essential for developing effective treatments and therapeutic strategies. In this study, we screened DEGs from the GSE120123 dataset and identified significant enrichment in terms related to inflammatory response, the IL-17 signaling pathway, and hematopoietic cell lineage. Prior research has highlighted the importance of these pathways in HCC. For example, Yang et al. [29] suggested that *Scutellaria barbata* may treat HCC by inhibiting core genes and blocking the IL-17 signaling pathway, thereby suppressing cancer cell proliferation and migration while inducing apoptosis. Similarly, Yu et al. [30] revealed that NF-kappaB plays a role in regulating Tec, a protein tyrosine kinase, suggesting its potential association with HCC development via the hematopoietic lineage pathway.

We conducted univariate Cox analysis on the TCGA-LIHC dataset to identify genes associated with prognosis. The TCGA-LIHC dataset was chosen for its comprehensive clinical data and rigorous quality control, making it a reliable resource for identifying prognostic genes. Overlapping genes from prognostically significant genes, DEGs of TCGA-LIHC, and DEGs from the GSE120123 dataset were screened for overall survival analysis, resulting in 57 genes with significant *P* values. Among the eight prognostic feature genes, CCDC170 and *F13B* have not been previously reported in HCC. *F13B*, in particular, was selected as a key gene after the prognostic risk model, expression, and survival analysis. Pathway enrichment analysis using GSEA revealed that *F13B* was significantly enriched in pathways related to nephrogenesis, the PPAR signaling pathway, and EGFR tyrosine kinase inhibitor resistance [21, 31, 32]. These pathways are relevant to HCC development. EGFR tyrosine kinase inhibitors (TKIs), for instance, have demonstrated clinical success in non-small cell lung cancer and show potent anti-proliferative effects on HCC cells. PPARs, as ligand-activated transcription factors, regulate various metabolic functions: PPARα in lipid metabolism, PPARβ/δ in fatty acid β-oxidation, and PPARγ in adipocyte triacylglycerol accumulation.

In summary, our findings suggest that the IL-17 signaling, EGFR inhibitor resistance, and PPAR pathways are crucial in HCC development. Identifying enriched genes within these pathways could provide promising directions for HCC diagnosis and therapy.

The cell cycle is a tightly regulated process that governs cell growth and division [33]. Key regulators include Cyclin B1, CDC2, CDC25, p21, and Cyclin D1. Cyclin B1 complexes with CDC2 to promote the transition from the G2 phase to mitosis, while CDC25 activates CDC2 by removing inhibitory phosphates [34]. p21, a CDK inhibitor, blocks Cyclin-CDK complexes, causing cell cycle arrest [35]. Cyclin D1 controls the transition from the G1 to the S phase, promoting cell cycle progression [36]. Together, these molecules ensure accurate cell division and proliferation. In HCC, dysregulation of the cell cycle is associated with poor prognosis and tumor progression. Zuo et al. [37] showed that SMYD2 expression in HCC correlates with aggressive tumor features and poor prognosis. Silencing SMYD2 inhibits cell proliferation and cycle progression, highlighting its potential as a prognostic biomarker. Similarly, Zuo et al. [38] demonstrated that TCP10L has tumor-suppressive properties in HCC, with its downregulation associated with advanced disease stages. Overexpression of TCP10L reduces colony formation, suggesting its potential as a diagnostic and therapeutic target. Additionally, Zou et al. [39] found that knockdown of lncRNA HUMT inhibits HCC proliferation and metastasis through the miR-455-5p/LRP4 axis, positioning it as a promising therapeutic target. Our in vitro experiments showed that *F13B* expression is low in HCC cell lines and tumor tissues. *F13B* overexpression inhibits invasion, migration, and proliferation of HCC cells while also affecting the expression of cell cycle proteins, leading to cell cycle arrest. These findings suggest that *F13B* could serve as an independent biomarker for HCC prognosis and a potential therapeutic target.

HUVECs are commonly used as a model for studying angiogenesis. By integrating experiments with both HCC cell lines and HUVECs, we aimed to explore *F13B*’s intrinsic effects on tumor cells and its impact on angiogenesis, a critical factor in tumor progression. This dual approach helps provide a comprehensive understanding of the interplay between cancer cells and endothelial cells, which is crucial for metastasis and the development of therapeutic strategies. VEGF is essential for angiogenesis as it promotes endothelial cell migration, proliferation, and tube formation. When exposed to VEGF, HUVECs activate VEGF receptors, triggering signaling cascades that upregulate angiogenesis-related genes, which are vital for wound healing and tissue regeneration [40]. Zhu et al. [41] showed that NT5DC2 knockdown reduces VEGF expression in colorectal cancer, hindering tumor growth and metastasis. Similarly, Zhu et al. [42] found that triptolide inhibits angiogenesis in anaplastic thyroid carcinoma by downregulating the NF-kappaB pathway in HUVECs and reducing VEGF expression in tumor cells. Studies by Zhu et al. [43] have shown that loss of IKBKE in glioblastoma downregulates VEGF through the AKT/FOXO3a pathway, inhibiting tumor growth and angiogenesis. CD31 is a marker for endothelial cells used to identify microvascular density, correlating with angiogenesis in tumors [44]. Increased CD31 expression indicates enhanced angiogenesis, often observed in aggressive tumors [42]. Our study found that *F13B* inhibits CD31 expression in HUVECs, suggesting it suppresses HCC angiogenesis. When HUVECs were exposed to VEGF, LDH release was reduced, and cell proliferation and invasion were enhanced. However, these effects were mitigated by *F13B* overexpression. LDH release is a marker of cell damage and cytotoxicity, and reduced LDH release upon VEGF induction indicates lower cell damage and higher viability, which aligns with increased proliferation and invasion. Knockdown of *F13B* in HCC cells increased *VEGFA* secretion and expression, while *F13B* overexpression inhibited HUVEC proliferation—an effect reversed by *VEGFA* overexpression. These findings suggest that *F13B* inhibits HCC angiogenesis by downregulating *VEGFA* expression, making it a potential therapeutic target in HCC.

The AKT/mTOR pathway is a key signaling cascade regulating cell growth, survival, and metabolism [45]. Activation starts when growth factors bind to cell surface receptors, leading to AKT phosphorylation. Activated AKT phosphorylates and activates mTOR, a central kinase that controls protein synthesis and cell growth [46]. The AKT/mTOR pathway integrates signals from upstream pathways like PI3K and PTEN, playing a vital role in cell homeostasis. This pathway is also important in cancer development. Zhou et al. [47] found that HSPA12B secretion from tumor-associated endothelial cells activates the PI3K/AKT/mTOR pathway in HUVECs, promoting an immunosuppressive microenvironment in head and neck squamous cell carcinoma. Zhang et al. [48] demonstrated that proanthocyanidins from Chinese bayberry leaves inhibit angiogenesis in HUVECs and induce a G1 cell cycle arrest in ovarian cancer cells by targeting the AKT/mTOR pathway. Similarly, Zhang et al. [49] found that AT-533, an Hsp90 inhibitor, impairs breast cancer growth and angiogenesis by inhibiting the HIF-1α/VEGF/VEGFR-2 and AKT/mTOR pathways in HUVECs. Our study found that VEGFR2, MMP2, MMP9, p-AKT, and p-mTOR expression increased in HUVECs after VEGF treatment, but this increase was mitigated by *F13B* overexpression. MMP2 and MMP9 are matrix metalloproteinases involved in breaking down extracellular matrix components, facilitating angiogenesis and metastasis. The attenuation of these increases by *F13B* suggests that it negatively regulates VEGF-induced signaling, particularly in the AKT/mTOR pathway, potentially reducing angiogenesis and metastasis in HCC.

The HIF-1α/VEGF pathway plays a key role in regulating angiogenesis and oxygen homeostasis [50]. Under hypoxic conditions, *HIF1A* stabilizes and moves to the nucleus, where it binds to hypoxia response elements (HREs) in the VEGF gene promoter, activating VEGF transcription [51]. VEGF then promotes angiogenesis by stimulating endothelial cell migration, proliferation, and enhancing cell permeability, all essential steps for new blood vessel formation. This pathway is critical for tumor growth and metastasis, making it a promising target for cancer treatment. Yu et al. [52] revealed that silymarin inhibits HCC cell proliferation under hypoxic conditions by targeting the HIF-1α/VEGF pathway, inducing apoptosis in HepG2 and Hep3B cells. Xiao et al. [53] showed that elevated γ-H2AX expression in HCC under hypoxic conditions is associated with tumor aggressiveness and poor prognosis, promoting angiogenesis through the EGFR/HIF-1α/VEGF pathways. Malami et al. [54] found that elevated γ-H2AX expression in HCC after liver transplantation is linked to tumor aggressiveness and poor prognosis, indicating its significance as a therapeutic target and prognostic marker. Our study showed that under hypoxic conditions, VEGF and HIF-1α levels significantly increased in HCC cells, along with cell viability. This increase was reduced by *F13B* overexpression, indicating that *F13B* plays a role in inhibiting hypoxia-induced HCC cell proliferation and VEGF/HIF-1α expression. These findings highlight *F13B*’s potential as a therapeutic target for inhibiting tumor growth and angiogenesis in HCC.

In conclusion, our study demonstrates the prognostic significance of *F13B*, with higher expression levels associated with better patient outcomes, including improved progression-free survival (PFS), disease-specific survival (DSS), and disease-free interval (DFI). Despite *F13B* being expressed at low levels in HCC, its potential as a therapeutic target is promising. Future research could explore small molecules [55, 56], pharmacological agents [57], or gene therapy [58] to upregulate *F13B* or inhibit its pathways in cancer cells. While our findings lay a foundation for these approaches, further studies are needed to assess the clinical potential of targeting *F13B*.

However, our research is not without limitations. Despite leveraging extensive public database data, the study’s retrospective nature and lack of access to raw patient samples limited our ability to perform additional molecular assays or clinical validations. The observed heterogeneity within the “normal” group may indicate underlying complexities not captured by database annotations, underscoring the need for future research involving direct patient samples. Additionally, the use of the GSE12023 dataset, although informative, may be limited compared to other datasets with larger sample sizes. Moving forward, we plan to utilize additional datasets to corroborate our findings, enhancing the generalizability and robustness of our conclusions.

## Conclusion

Our bioinformatics analysis of HCC-related datasets identified *F13B* as a prognostic gene with therapeutic potential. Functional experiments showed that *F13B* overexpression suppressed HCC proliferation, migration, and invasion, while its knockdown promoted these processes. Additionally, *F13B* inhibited angiogenesis by downregulating *VEGFA* and key signaling pathways, including the AKT/mTOR pathway. *F13B* also attenuated VEGF-induced cytotoxicity and reduced LDH release, suggesting it reduces cell damage. Mechanistically, *F13B* negatively regulates the HIF-1α/VEGF pathway, especially under hypoxic conditions, highlighting its potential as a therapeutic target in HCC. Our study underscores the importance of *F13B* not only as a prognostic marker but also as a target for future therapeutic interventions in HCC.

## Supplemental data

## Data Availability

The datasets used and/or analyzed during the study are available from the corresponding author upon reasonable request.
